# Informed mutation of western equine encephalitis virus to heparan sulfate binding: Implications for rational design of alphavirus live attenuated vaccines

**DOI:** 10.1371/journal.ppat.1013941

**Published:** 2026-02-09

**Authors:** Tetyana Lukash, Shishir Poudyal, Theron C. Gilliland, Thomas Klose, Chengqun Sun, Shauna N. Vasilatos, Jessica L. Farren, Long Kwan Metthew Lam, Richard J. Kuhn, William B. Klimstra

**Affiliations:** 1 Center for Vaccine Research and Department of Immunology, University of Pittsburgh, Pittsburgh, Pennsylvania, United States of America; 2 Department of Biological Sciences and Purdue Institute of Inflammation, Immunology, and Infectious Disease, Purdue University, West Lafayette, Indiana, United States of America; University of North Carolina at Chapel Hill, UNITED STATES OF AMERICA

## Abstract

Encephalitogenic alphaviruses are mosquito-borne viruses that can cause fatal disease in humans and equines. Currently, there are no licensed vaccines or antiviral treatments for these infections. Western equine encephalitis virus (WEEV) is a member of this group that had not produced a human infection in over a decade. However, an outbreak of WEEV encephalitis in humans and equines was reported recently in South America, indicating a need for additional countermeasures. Blind passage approaches to generation of RNA virus live attenuated vaccines (LAVs) frequently result in acquisition of positively charged amino acid mutations that confer heparan sulfate (HS) binding and that are attenuating factors in resultant LAVs. To develop an informed approach for creation of alphavirus LAVs, we have utilized the WEEV McMillan (McM) strain as an HS weak/non-binding platform into which we have placed positively charged amino acid substitution mutations at positions in the E2 glycoprotein previously shown to confer HS-dependent infection upon other alphaviruses. This approach yielded four mutants with high efficiency HS binding and avirulence in mice, which were further subjected to yield optimization by *in vitro* selection of second-site mutations. Interestingly, the original mutations concomitantly increased HS interactions and reduced infection promoted by VLDLR and PCDH10 protein receptors, while the second site mutations improved infectivity mediated by VLDLR. Further, we report a newly generated 4.1Å cryo-EM reconstruction of WEEV McM strain into which we have mapped the mutations to provide an E2 glycoprotein domain-based representation of receptor binding site location.

## Author summary

Using the western equine encephalitis virus (WEEV) McMillan strain, we introduced rationally selected, positively charged amino acid substitution mutations into the E2 glycoprotein to promote heparan sulfate (HS)-dependent entry and attenuation. Several mutations resulted in increases in HS binding and mouse attenuation and, surprisingly, reduced engagement of known WEEV protein receptors VLDLR and PCDH10. Additional passage of mutant viruses yielded second-site mutations that preserved HS binding and attenuation, and increased engagement with protein receptors. Structural localization of mutations in a new cryo-EM model of WEEV revealed clustering within E2 functional domains. These findings provide a structure-informed framework for engineering receptor-specific attenuation and optimizing alphavirus LAV candidates for improved safety and production characteristics.

## Introduction

Alphaviruses are mosquito-borne members of the *Togaviridae* family of RNA viruses that can cause human disease ranging from febrile illness to arthritis and/or fatal encephalitis. Currently, there are no licensed vaccines or effective therapeutics to combat alphavirus infections. Western equine encephalitis virus (WEEV) is an encephalitic alphavirus historically confined to the New World that can cause fatal encephalitis in humans and equines. WEEV circulates naturally in birds and is transmitted to equines and humans through the bite of an infected mosquito vector. The virus is a recombinant derived from Sindbis-like and eastern equine encephalitis virus (EEEV) ancestors [1,2].

Until recently, human cases of WEEV had not been seen in the Americas for almost two decades, but during November 2023 - April 2024, an outbreak of equine (2548 cases in Argentina and Uruguay) and human (217 confirmed cases, including 12 fatalities) infections occurred in the South American countries of Argentina, Paraguay and Uruguay [3,4]. Prior to this event, the last outbreak in equines was reported in Mexico in 2019 (Promed archive 20190406.6407111 [5]) and the most recent human case was in Uruguay in 2009 [6]. This raises the possibility of broader emergence of WEEV and reinforces the need for advances in vaccines and therapeutics targeting the encephalitic alphaviruses.

Blind cell culture passage of virulent virus strains has historically been used with many viruses, including alphaviruses, to create live attenuated vaccines (LAV) [7–11]. Many of these vaccine strains exhibit enhanced binding to cell surface glycosaminoglycans *versus* parental strains, most frequently interacting with heparan sulfate (HS) chains on cellular proteoglycan receptors. Enhanced HS binding is typically associated with acquisition of negatively to positively charged amino acid (aa) substitution mutations in attachment proteins that, presumably, interact directly with negatively charged HS chains [12]. Mutations that confer efficient HS binding are often primary attenuating factors in LAVs derived by blind passage and are found in LAVs derived from Yellow Fever Virus (17D) [13]), Japanese encephalitis virus (SA14-14-2) [14], chikungunya virus (CHIKV) (181/25) [15], dengue virus (recombinant ChimeriVax vaccine) [16,17], and Venezuelan equine encephalitis virus (VEEV) (TC83) [18]. Informed optimization of the cell binding and replication phenotypes of viral structural proteins could be useful for the development of high titer, highly immunogenic LAVs.

Here, we demonstrate that the wild type (WT) WEEV McMillan (McM) strain does not interact strongly with HS on cells and its infection *in vitro* is essentially HS-independent. Initial attempts to derive a HS-binding mutant of WEEV by passage on BHK cells generated a positively charged substitution mutation, but surprisingly, it did not increase HS-dependence of the virus *versus* the wild type. Using WEEV as a HS binding-negative platform, we have attempted to develop an informed method for alphavirus LAV design, by introducing into analogous positions of the E2 glycoprotein, previously published and newly identified negatively-positively charged amino acid cell culture-adaptive substitution mutations derived through cultured cell passage of arthritogenic and encephalitogenic alphaviruses including, Sindbis (SINV), CHIKV, Getah (GETV), and VEEV. Furthermore, these mutations were then mapped into a newly generated cryo-electron microscopy (cryo-EM) reconstruction of the WEEV McM strain.

Most of the mutations, including those in the flexible region preceding domain A, domain A, domain B, and the domain A-B hinge region of E2, conferred HS-dependent infectivity upon WEEV. Four mutations that conferred significantly increased HS-dependent infectivity *versus* the parental McM strain and were avirulent in mice after subcutaneous infection were chosen for further development. We addressed vaccine scale-up issues, such as detrimental effects of informed mutation placement on virus particle infectivity by using *in vitro* selection of second site mutations to increase replication efficiency and particle yields, while retaining HS-mediated infection dependence. Notably, all of the chosen first site mutations enhancing HS-dependent infectivity also decreased infectivity promoted by VLDLR and PCDH10, recently identified WEEV protein receptors [19–21], while all of the second site mutations significantly improved infectivity mediated by VLDLR but not PCDH10. In a mouse model of subcutaneous LAV vaccination, optimized HS-dependent viruses were largely avirulent and elicited protective neutralization titers, suggesting that informed placement of attenuating mutations in the alphavirus E2 has utility in LAV design, likely due to the conserved three-dimensional domain structure of the viral spike heterodimer and potential promiscuity of the HS-protein interaction.

## Results

### In vitro passage of wild type SINV-WEEV did not select E2 glycoprotein mutations conferring HS binding

Positively charged residues, Arg and Lys, are crucial for the interaction of proteins with HS [22,23], and these residues are frequently found in LAVs derived from RNA viruses (reviewed in [12]). We began these studies by passaging WT WEEV on BHK-21 fibroblasts to determine if mutations conferring HS binding could be selected with this virus, as had been observed with other alphaviruses [15,18,24]. We constructed a SINV-WEEV-eGFP chimera virus in which the nonstructural protein genes (nsP1-4) were derived from Sindbis virus (SINV, strain TR339) [25] and the structural protein genes (C, E3, E2, 6K, E1) from WEEV strain McMillan [26,27], along with enhanced green fluorescent reporter gene (eGFP) [15], which allowed identification of changes in cell infection rates and for experiments to be performed in BSL-2 biocontainment.

**Fig 1:**
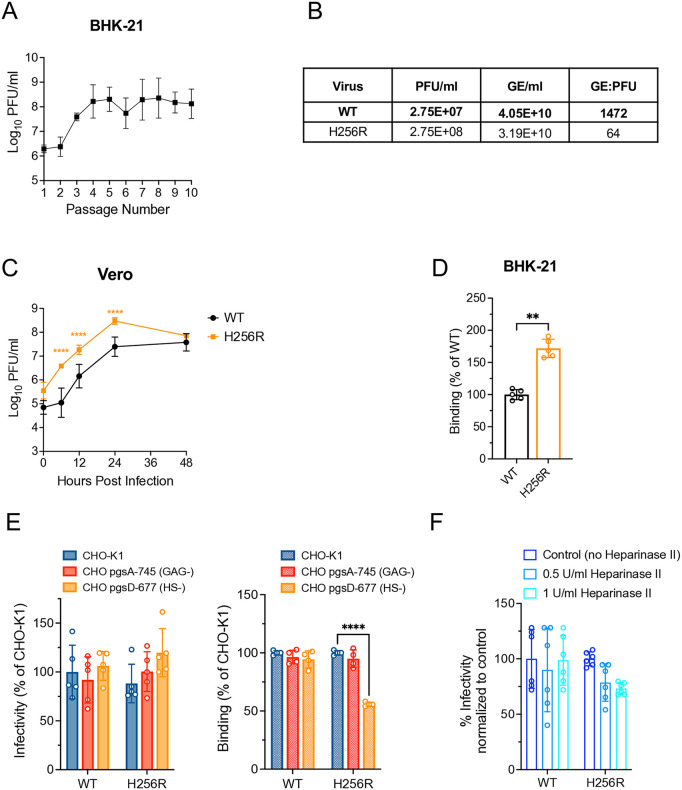
In vitro passage of wild type SINV-WEEV did not select mutations conferring HS binding in the E2 glycoprotein. **(A)** SINV-WEEV-eGFP virus stock was serially passaged 10 times on BHK-21 cells. **(B)** Concentrations of PFU/ml, GE/ml and GE:PFU ratios in viral stocks. Infectious viral titers and genomic RNA levels in culture medium were determined by plaque assay on BHK-21 cells and RT-qPCR, respectively. The genomic RNA:PFU ratios were calculated to indicate virion infectivity. **(C)** SINV-WEEV-eGFP E2 mutants replication *in vitro* in Vero cells. Vero cells were infected with equal genomes of WT and E2-H256R mutant corresponding to a multiplicity of infection of 1 PFU for WT SINV-WEEV-eGFP. Media were collected at the indicated times post infection, and infectious titers were determined by plaque assay on BHK-21 cells. **(D)** Relative binding of SINV-WEEV-eGFP WT and E2-H256R for BHK-21cells. Diluted viruses at an MOI of 5 were added to indicated cells and incubated for 45 min at 4 °C. Unbound virions were removed by washes with cold PBS, and ddPCR was used to assess the amount of bound viral particles. **(E)** Relative infectivity and binding of the indicated SINV-WEEV-eGFP viruses on CHO-K1, GAG-deficient pgsA-745 and HS-deficient pgsD-677 cells. For the infectivity assay, viruses were diluted and assayed for plaque formation on indicated cell monolayers. eGFP expressing foci were enumerated with a fluorescence microscope. Binding assay was performed as described above. Percent infectivity and binding were normalized to CHO-K1, which was set to 100%. **(F)** Effect of heparinase II treatment on plaque formation by the chimeric viruses. Confluent BHK-21 cell monolayers were treated with heparinase II at the indicated concentrations. After washing with PBS, the cells were infected with diluted viruses, and plaque assays were performed. Error bars are standard deviations. Significance (*P* values) was estimated by (C) two-way ANOVA with Bonferroni’s post-test, (D) Mann-Whitney test, (E,F) two-way ANOVA with Dunnett’s post-test: *P < 0.05, **P < 0.01, ***P < 0.001, **** P < 0.0001.

Infectious titers in supernatants, harvested after each passage, gradually increased from 2.0 x 10^6^ PFU/ml at starting passage 1 (P1) to 2.78 x 10^8^ PFU/ml at P4 and reached the highest titer – 4.55 x 10^8^ PFU/ml at P8 (Fig 1A). These data suggested adaptation of the population to more efficient replication in cell culture within 10 passages. The E2 gene from virus populations at P4, P7 and P10 was sequenced to identify passage-associated mutations. By passage 4, we detected a polar to positively charged substitution from His to Arg in the E2 B-C domains β-ribbon connector at position 256. This mutation was incorporated into the SINV-WEEV-eGFP chimera cDNA by site-directed mutagenesis to generate the SINV-WEEV E2 H256R-eGFP mutant virus. The mutant virus was viable, and the genome to PFU ratios (GE:PFU) demonstrated ~20-fold decrease compared to WT (Fig 1B). This suggested that individual particles of the H256R mutant virus were more infectious for BHK cells than the WT.

We compared the replication of SINV-WEEV E2 H256R to the WT virus in Vero cells (Fig 1C). There was a significantly higher yield of infectious SINV-WEEV E2 H256R than WT with peak titers of 3.0 x 10^8^ PFU/ml for the mutant vs 3.4.0 x 10^7^ PFU/ml for WT at 24 h post infection; although, some of the mutant’s increase in infectious titer may reflect higher infectivity for the BHK indicator cells in the plaque assay. BHK-21 cell binding showed ~2-fold increase in attachment efficiency of E2 H256R mutant compared to the WT virus (Fig 1D). In order to examine the effect of E2 H256R mutation on HS-dependent infection, we compared infectivity and binding of the mutant and WT viruses for wild-type CHO-K1 cells and two mutant CHO cell lines: pgsA-745 and pgsD-677 (Figs 1E and S2). CHO-K1 cells express approximately 70% heparan sulfate and 30% chondroitin sulfate, while pgsD-677 cells are deficient in heparan sulfate, but express three- to four-fold-higher levels of chondroitin sulfate, and pgsA-745 cells are deficient in all glycosaminoglycans (GAGs) [28,29]. Lower infection and binding rates for the GAG- or HS-deficient cells imply dependence on GAGs or HS for infection [22]. The infectivity of E2-H256R with CHO-K1 or GAG-negative or HS-negative cells were similar to the WT indicating little dependence on GAGs for infectivity (Fig 1E). Binding to pgsA-745 cells was similar to WT cells, but significantly reduced in pgsD-677, suggesting that cell surface GAGs other than HS may be involved in cell binding but not cell infection.

We also examined the infectivity of the E2 H256R mutant for BHK-21 cellsstrike treated with microbial heparinase II (HepII), which specifically cleaves heparin and heparan sulfate glycosaminoglycans regardless of their sulfation patterns [30,31]. The infectivity of the E2 H256R mutant was not significantly reduced on BHK-21 cells treated with heparinase II (Fig 1F), confirming that H256R does not enhance dependence upon HS for infection. Thus, the mutation may affect another receptor attachment/entry pathway used by WEEV [19–21].

### Informed design of HS binding WEEV LAV candidates

To develop an informed method for alphavirus LAV design and compare E2 domain functions between divergent alphaviruses, we introduced 16 previously published and newly identified negatively-positively charged amino acid mutations either from natural HS binding sites or identified after cell passage of arthritogenic and encephalitogenic alphaviruses, including SINV, CHIKV, GETV, WEEV and VEEV, into analogous positions in the WEEV McM E2 protein (S1 Table). The mutations were selected based on the ability of analogous residues to confer efficient HS binding on other alphaviruses. Occasionally, a negatively charged amino acid was not present at the analogous WEEV E2 position, in which case, a similarly positioned amino acid was mutated. Eleven negatively charged residues were substituted: D to K at E2-4, E2-70, E2-72, E2-74, E2-77, E2-78, E2-156; or D to R at E2-60; E to K at E2-182; E to R at E2-81 and E2-160. Four neutrally charged amino acids were substituted with a positively charged: S to R at E2-1, E2-114; G to R at E2-172 and E2-209. One mutant with positively to positively charged amino acid substitution K to R at E2-254 was selected as this exchange at an analogous position improved GETV attachment by enhancing binding to HS [32]. Viruses with randomly selected E2 negative-positively charged aa substitutions in the E2 A, A-B β-ribbon or B domains: D109K, E138K and D205K were also created (Table 1). Each E2 protein mutation was incorporated into the WT McM and SINV-WEEV-eGFP chimeric virus cDNA clones as described in Materials and Methods. The genotypes of the viruses used in this study are summarized in Table 1. Virus stocks were made and the presence of the desired mutation within the rescued virus stock was confirmed by RNA sequencing.

**Table 1:** WEEV E2 positive charge substitution mutations.

E2 substitution mutation	E2 Domain	Specific infectivity (GE:PFU)		Source^1^		Reference
		BHK-21 cells	CHOK1 cells	E2 mutant	E2 WT	
**WT**		**1409**	**1663**			[26]
S1R	N-linker	1552	3206	SINV		[24]
D4K	N-linker	145	196	VEEV		[18]
D60R/V61T	A	834	301	SINV		(This manuscript)
D70K	A	1363	3538	SINV	EEEV	[24,33]
D72K	A	527	1514	EEEV		[34]
D74K	A	3493	1956		EEEV	[33]
E77K	A	2092	312403	VEEV	EEEV	[18,33]
D78K	A	5104	46366	CHIKV		[15]
E81R	A	1147	2051	CHIKV		[15]
S114R	A	2974	1024	SINV		[24]
D156K	A-B β-ribbon	1048	4914		EEEV	[35]
E160K	A-B β-ribbon	101	833	CHIKV		[15]
G172R	B	1891	1103	SINV		[36]
E182K	B	7491	7128	WEEV		[37]
G209R	B	10680	49171	VEEV SINV		[18,36]
K254R	B-C β-ribbon	4483	1916	GETV		[32]
D109K	A	2248	1941			–
E138K	A-B β-ribbon	789	8548			–
D205K	B	727	765			–

^1^ Indicates whether positively charged residue comes from a mutant or wild type virus.

To examine the effects of the mutations on virus infectivity per particle, a genome to PFU ratio (GE:PFU) for each SINV-WEEV virus stock was determined for BHK-21 and CHO-K1 cell lines (Table 1). Wild type SINV-WEEV-eGFP had mean GE:PFU ratios of 1,409 and 1,663, on BHK and CHOK1 cells, respectively. In this assay, a lower GE:PFU ratio implies greater infectivity per virus particle. The lowest GE:PFU ratios of 1.7 to 14-fold decrease compared to WT were observed for mutants D4K, D60R/V61T, E160K and D205K on both BHK-21 and CHOK1 cell lines. Mutant D72K had 2.7-fold decrease versus WT for BHK-21 cells and no difference for CHO-K1. Mutants D74K, D77K, D78K, E182K, G209R and K254R exhibited GE:PFU ratios of 1.2 to 188-fold higher compared to wild type virus in both cell lines. Mutants S1R, D70K, D156K and E138K did not show a significant difference for BHK-21 cells but had 2–5-fold higher GE:PFU ratios in CHO-K1 cells. No significant difference was found with either cell line for mutants E81R, G172R and control E2 mutant D109K. These results suggest that five mutations out of nineteen analyzed: D4K, D60R/V61T, D72K, E160K and D205K decrease the virus particle to PFU ratio on both cell types, indicating a general increase in infectivity. We expected that mutations increasing HS binding would decrease particle to PFU ratios, as is common with other viruses (Reviewed in [12]). However, such decreases may be a result of particular mutations on other factors, such as particle stability.

### Cryo-EM reconstruction of the SINV-WEEV McM strain and mutational mapping

**Fig 2:**
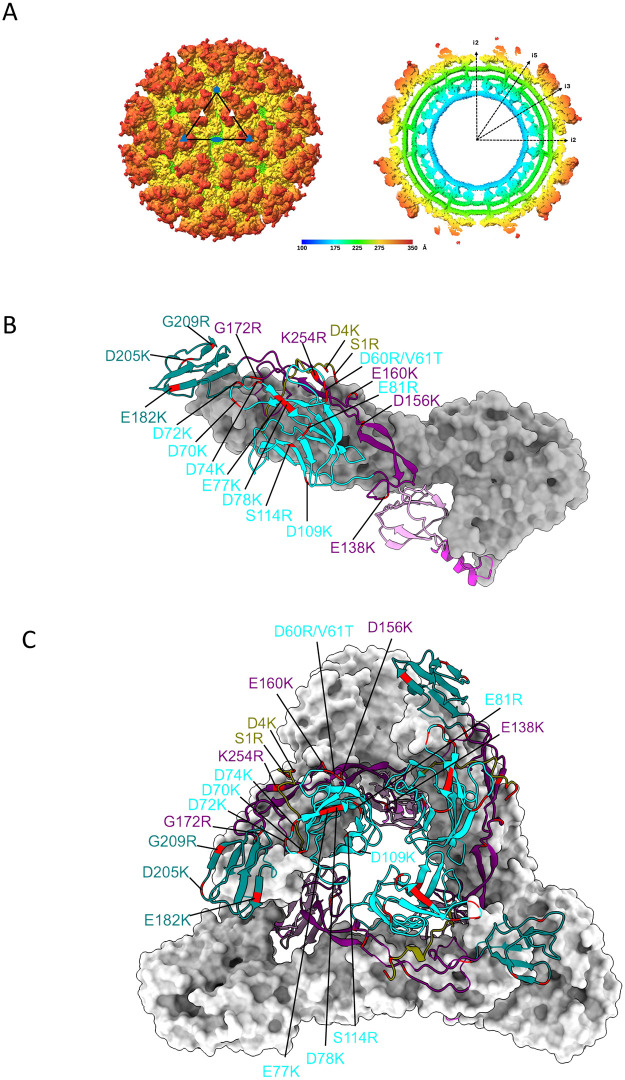
Structural organization of SINV-WEEV McM strain and mutational mapping of HS-binding residues on a ribbon model of the WEEV McM E2 trimer. **(A)** Left panel, Icosahedral reconstruction of SINV-WEEV McM strain. The black triangle represents one asymmetric unit. The icosahedral five-, three-, and two-fold symmetry axes are shown with blue pentagon, triangle, and oval, respectively. The q3 spikes are indicated with white triangles. Right panel*,* central cross-section of the volume viewed down an i2-axis. Other symmetry axes are labeled. The color key represents the radial coloring of the volume in Å. **(B)** Three-dimensional model of the E2-E1 heterodimer of WEEV McM and **(C)** trimeric spike complex of E2-E1 heterodimers. Ribbon drawings show the subdomain distribution in E2 are colored according to the following scheme: domain A – cyan, domain B – teal, domain C – plum, domain D – magenta, β-ribbon – purple, N-linker - olive. The E1 glycoprotein is in grey. Locations of the mutations are highlighted in red. The figures were made using UCSF ChimeraX software.

A 4.1 Å three-dimensional cryo-EM reconstruction of the SINV-WEEV McM chimera was generated, demonstrating a general structural conservation with other alphaviruses (Fig 2). The E1 fusion protein, whose ectodomain is divided into domains I, II, and III, is positioned approximately parallel to the viral membrane. In contrast, the E2 protein, which facilitates receptor engagement and attachment, consists of domains A, B, and C in its ectodomain and is connected by a β-ribbon connector (Fig 2). The SINV-WEEV icosahedral reconstruction revealed a prominent glycan density corresponding to N199, accounting for approximately 52.7e^6^ Å^3^ in volume, suggesting the incorporation of a complex glycan from the mammalian cell line BHK-21 [38].

To provide a detailed domain-based representation of HS binding, an asymmetric unit was further locally refined using CryoSPARC [39], resulting in a 3.2 Å local reconstruction. This locally reconstructed map was subsequently used for a rigid body fitting of an asymmetric unit (PDB ID: 8DEE [40]), which was then manually rebuilt in COOT [41] and refined in Phenix [42] against the locally reconstructed map to obtain the coordinates for the asymmetric unit of the WEEV McM strain. The newly modeled structure was utilized for informed insertion of residues implicated in HS binding and attenuation of other alphavirus vaccine vectors (Fig 2).

### Effects of E2 glycoprotein mutations on the HS dependence of WEEV infectivity and binding

**Fig 3:**
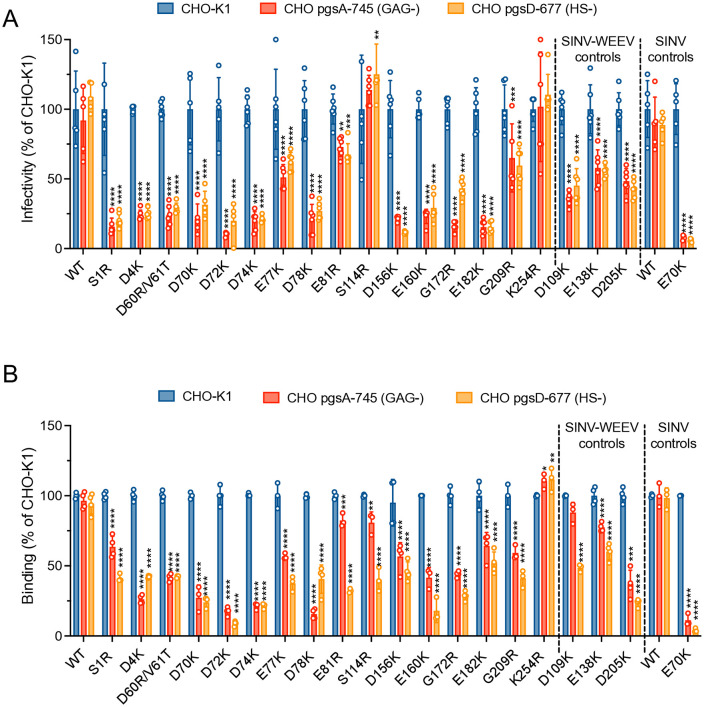
Relative infectivity and binding of SINV-WEEV E2 mutant viruses on CHO-K1, GAG-deficient pgsA-745 and HS-deficient pgsD-677 cells. **(A)** For infectivity assay, wild-type and mutant viruses were serially diluted and assayed for plaque formation on the indicated cell monolayers. eGFP expressing foci were enumerated with a fluorescence microscope at 36 h post infection. **(B)** For binding assay, monolayers of each cell type were incubated with the indicated viruses at an MOI of 5 for 45 min at 4 °C and washed with PBS supplemented with 1% FBS to remove unbound virions. Percent infectivity/binding was normalized to CHO-K1, which was set to 100%. SINV TR339 and E2-E70K mutant are included as HS–independent and HS–dependent controls, respectively. The data are representative of two independent experiments; each performed in duplicate or triplicate. Column heights indicate mean values, and error bars denote SD. Statistical analysis: two-way ANOVA with Dunnett’s post-test: *P < 0.05, **P < 0.01, ***P < 0.001, **** P < 0.0001, compared to CHO-K1. Only significant comparisons are represented; all others were non-significant.

The effect of the WEEV E2 protein mutations on HS-dependent infectivity and binding was evaluated using eGFP expressing chimeric SINV-WEEV virus infection of wild-type CHO-K1 and the GAG- or HS- CHO mutant cells. Control viruses included SINV TR339 (minimal HS binding, [24]) and the TR339 E2 K70 cell-adapted mutant (efficient HS binding [24]). SINV-WEEV WT, S114R, K254R and the SINV TR339 control virus infected wild-type CHO-K1 cells as well as the CHO GAG-deficient pgsA-745 and HS-deficient pgsD-677 cells, indicating that infection was not dependent on HS expression (Fig 3A). In contrast, all other E2 glycoprotein mutant viruses exhibited some dependence upon GAGs. The infectivity of S1R, D4K, D60R/V61T, D70K, D72K, D74K, D78K, D156K, E160K, G172R, E182K for CHO-K1 cells all showed significant dependence upon GAGs (P < 0.0001), evidenced by ≥ 75% reduced infectivity on CHO pgsA–745 cells with the highest (90%) reduction observed for the D72K mutant. E77K exhibited a significant (P < 0.0001) but intermediate GAG dependence of ~50% reduced infectivity. Mutants E81R and G209R had only a ~ 30% reduction in infection efficiency (P < 0.01 and P < 0.001, respectively). Control mutants D109K, E138K and D205K, which reside in either the E2 A, A-B connector or B domains (Table 1), surprisingly showed, respectively, 65%, 42% and 52% infectivity decreases (P < 0.0001) on the GAG negative cells. This suggests that small changes in particle surface charge, regardless of E2 position, can affect cell engagement. Alternatively, these mutations may have an effect on binding to non-HS receptors. Infectivity results on the HS-deficient pgsD-677 cells were generally similar to the pgsA-745 cells (Fig 3A), suggesting that HS was the primary ligand influencing infectivity differences.

Consistent with the infectivity, SINV-WEEV-eGFP WT, S114R and K254R, and the SINV TR339 control showed similar binding efficiency with all three cell lines, again implying little interaction with HS. While the S114R mutant infected wild-type CHO-K1 cells, it showed a small GAG dependence in binding to the GAG negative cells (~17% reduction, P < 0.01). Binding of D4K, D70K, D72K, D74K, D78K, E160K, G172R and SINV TR339 E70K control to the GAG-deficient pgsA-745 and HS-deficient pgsD-677 cells was reduced by 75–90% compared with CHO-K1 cells (Fig 3B). The binding of all other mutants exhibited moderate GAG dependence, reflected by a significant decrease in binding to the GAG negative cells. As with infectivity, E138K and D205K exhibited significant binding reductions (P < 0.0001) on both mutant CHO cell types, and D109K was significantly reduced on pgsD-677 (P < 0.0001), but not on pgsA cells. As with infectivity (Fig 3A), binding of most mutant viruses to the HS-deficient pgsD-677 cells was similarly reduced to the pgsA-745 cells (Fig 3B).

### Growth kinetics of E2 mutant viruses

**Fig 4:**
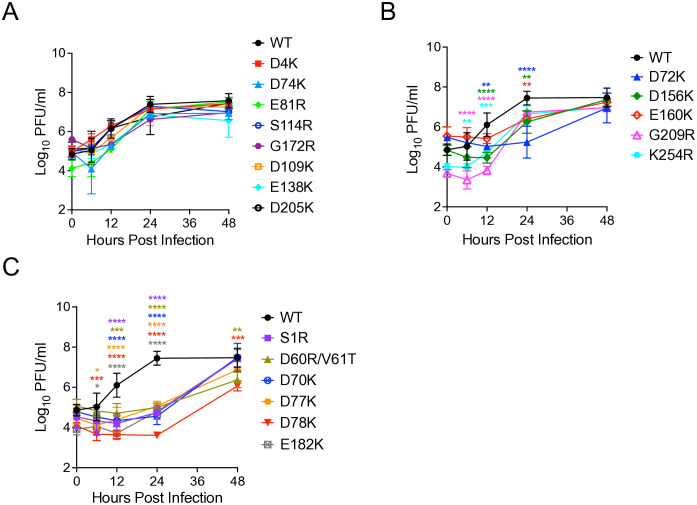
SINV-WEEV E2 mutants replication *in vitro* in Vero cells. Vero cells were infected with equal genomes of WT SINV-WEEV and E2 mutants corresponding to a multiplicity of infection of 1 PFU for WT. Media were collected at the indicated times post infection, and infectious titers were determined by plaque assay on BHK-21 cells. Data is represented as a geometric mean and error bars represent the standard deviation (SD). Each data point is from 3 independent experiments that were performed in duplicate. **(A)** Mutants with replication efficiency similar to WT. **(B)** Mutants with slightly delayed replication rates compared to WT. **(C)** Mutants with significantly delayed PFU production compared to WT. Significance determined by two-way ANOVA with Dunnett’s multiple-comparison test, *P < 0.05, **P < 0.01, ***P < 0.001, **** P < 0.0001. Only significant comparisons are represented, all others were non-significant.

To determine whether or not E2 mutations affected virus growth *in vitro* (a cumulative measurement of several factors including replication efficiency, particle yield and stability and/or infection efficiency), Vero cells were infected with equal genomes of each SINV chimera, equivalent to an MOI of 1 BHK PFU for WT SINV-WEEV, to compensate for differences in specific infectivity (GE:PFU ratio). Comparison of growth kinetics of the LAV candidates identified three main groups (Fig 4): 1) mutants with replication efficiency as measured by plaque assay similar to WT: D4K, D74K, E81R, S114R, G172R, D109K, E138K, D205K (Fig 4A); 2) mutants with slightly delayed PFU production compared to WT: G209R at 6 and 12 hpi (P < 0.0001), D156K at 12 and 24 hpi (P < 0.0001 and P < 0.01, respectively), K254R at 6 and 12 hpi (P < 0.01 and P < 0.001, respectively), D72K at 12 and 24 hpi (P < 0.01 and P < 0.0001, respectively), and E160K at 24 hpi (P < 0.01) (Fig 4B); and 3) mutants with dramatically delayed virion production at 6 hpi (D77K and E182K, P < 0.05; D78K, P < 0.001), 12 hpi (all mutants, P < 0.001 - P < 0.0001) and 24 hpi (all mutants, P < 0.0001), and 250–8000-fold lower titers at 24hpi compared to WT (Fig 4C). Remarkably, all the mutants except D60R/V61T and D78K (P < 0.01) reached a maximum titer that was not significantly different from WT at 48 hpi. These results demonstrate that most of the LAVs are capable of growth similar to WT WEEV *in vitro* and could be considered for further testing as LAV candidates.

### Effects of mutations on morbidity and mortality of infected mice

**Fig 5:**
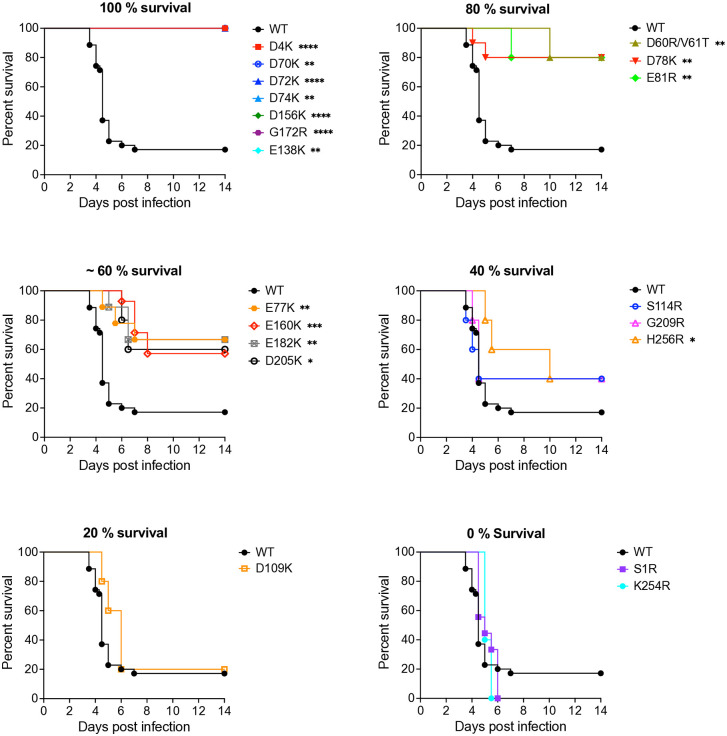
Mouse survival after subcutaneous LAV candidates infection. CD-1 mice were infected subcutaneously in rear footpad with the same dose of 10^3^ PFU of parental WEEV and the designed LAV candidate. Experiments included 4-5 animals per group. The significance between survivals of mice infected with WT WEEV and mutants was estimated using the Log-rank (Mantel Cox) test; *P < 0.05, **P < 0.01, ***P < 0.001, **** P < 0.0001.

To begin to examine the ability of the LAV candidates to function as attenuated and immunogenic vaccine vectors, we examined the virulence of the positive charge mutants placed into a parental WEEV cDNA clone in comparison to wild type WEEV following a primary subcutaneous (s.c.) infection of mice (Table 2). Four- to five-week-old CD-1 mice were infected with the same dose, 1000 PFU, of each virus and monitored daily for morbidity and mortality. Following s.c. infection, WT, control E2 mutant D109K, and S1R and K254R LAV candidates showed limited survival of 0–20% and mean survival time (MST) of 4.9 – 7 days (Fig 5). The S114R, G209R, and H256R mutants yielded 40% survival and an MST of 7.8 – 9.6 days, which was statistically significant versus WT for the H256R mutant (P < 0.05). All other LAVs were significantly attenuated compared to WT. Infection with E77K (P < 0.01), E160K (P < 0.001), E182K (P < 0.01), D205K (P < 0.05) yielded 60% survival and an MST of 10.8 – 11.3 days and D60R/V61T, D78K, E81R yielded 80% survival and an MST of 12.1 – 13.2 days (P < 0.01). Subcutaneous infection with nine of the 19 mutants, including the control virus E138K, resulted in 100% survival. Mutants with a high degree of dependence upon HS for infectivity and binding, and no mortality after subcutaneous infection (D4K, D72K, D156K and G172R), were downselected for further analysis.

**Table 2:** Mortality rates and mean survival time (MST) of the LAV candidates in outbred CD-1 mice after primary subcutaneous infection.

Virus	Survival (%)	MST ± SD^a^ (days)
WT WEEV McM	3/30 (10)	5.7 ± 3.5
S1R	0/9 (0)	4.9 ± 0.9
D4K	10/10 (100)	N/A
D60R/V61T	4/5 (80)	13.2 ± 1.8
D70K	5/5 (100)	N/A
D72K	19/19 (100)	N/A
D74K	5/5 (100)	N/A
E77K	6/9 (66.7)	11.1 ± 4.4
D78K	8/10 (80)	12.1 ± 4.0
E81R	4/5 (80)	12.6 ± 3.1
S114R	2/5 (40)	7.8 ± 5.7
D156K	10/10 (100)	N/A
E160K	8/14 (57)	11.1 ± 3.5
G172R	14/14 (100)	N/A
E182K	6/9 (66.7)	11.3 ± 4.1
G209R	2/5 (40)	8.0 ± 5.5
K254R	0/5 (0)	5.0 ± 0.0
H256R	2/5 (40)	9.6 ± 4.5
D109K	1/5 (20)	7.0 ± 4.0
E138K	5/5 (100)	N/A
D205K	3/5 (60)	10.8 ± 4.4

^a^SD: Standard deviation.

### Passaging of candidate LAVs *in vitro* increases the apparent yield of virions while retaining HS binding phenotypes

**Fig 6:**
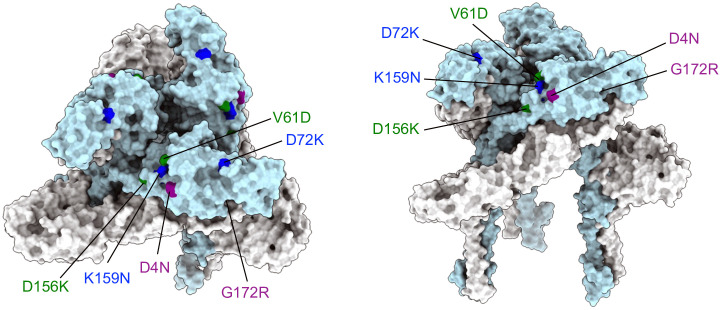
Three-dimensional model of the WEEV McM trimeric spike complex of E1/E2 heterodimers. Surface representation of WEEV McM spike trimer with E2 in light blue and E1 in grey. Locally reconstructed map obtained using newly generated 3.2Å cryo-EM reconstruction of WEEV McM strain. Location of the double mutations is highlighted in blue – D72K-K159N, green – D156K-V61D, and purple - G172R-D4N.

While original downselected mutants: D4K, D72K, D156K and G172R exhibited lower or similar GE:PFU ratios to the WT (Table 3), virus stocks infectious titers on BHK-21 cells were ~10 – 50-fold lower than the WT, and the replication of D72K and D156K mutants in Vero cells was delayed compared to WT, suggesting a detrimental impact of the mutations on virus particle stability and/or yield. To address this potential issue with LAV production scale-up, we attempted to use cell passage to select second site mutations that increased plaque titers and lowered GE:PFU ratios to levels similar to or lower than WT WEEV. Downselected SINV-WEEV chimeric E2 mutant viruses: D4K, D72K, D156K and G172R were passaged serially 10 times on BHK-21 cells. The E2 gene was then sequenced from virus populations at P5 and P9 to identify mutations that had accumulated during cell culture passage (Table 3 and S1 Fig). Sequences of all resultant viruses maintained the original mutations after replication in BHK-21 cells. By passage 10, we detected K159N substitution in the passaged D72K mutant supernatant, V61D in the passaged D156K mutant and D4N in the passaged G172R mutant. No additional mutations were detected with the passaged D4K mutant, which exhibited the lowest GE:PFU ratio originally. Interestingly, all three second site mutations were charged aa substitutions; however, none were from negatively to positively charged residues, as is typical for *in vitro-*selected mutations that confer HS binding. Second site mutations were incorporated in combination with original mutations into the SINV-WEEV-eGFP chimera and parental WEEV McM–based mutant cDNAs creating the double mutants: D72K-K159N, D156K-V61D and G172R-D4N. Sequencing of the E2 gene in the rescued viruses confirmed the presence of introduced mutations. Second site mutations were placed into the Cryo-EM reconstruction of WEEV McM alongside each original downselected mutation (Fig 6). Residues E2-4, E2-61, E2-72, mapped in Domain A, E2-156, E2-159 in the β ribbon connector between Domains A and B, and E-172 in domain B. Residues at positions 156 and 61 in the mutant D156K-V61D were located in close proximity on the virion surface and may participate in a conformationally determined HS/receptor-binding site.

**Table 3:** Downselected and BHK-21 cells passage-derived WEEV McM E2 positive charge mutants.

E2 substitution -selected mutation	E2 Domain	Stock titer (PFU/ml, BHK-21)	GE:PFU	
			BHK-21	CHO-K1
WT		2.75E + 07	1409	1663
D4K	A	2.45E + 06	145	196
D72K	A	1.35E + 06	527	1514
D72K-K159N	A/ A-B β-ribbon	4.45E + 07	221	119
D156K	A-B β-ribbon	6.00E + 05	1048	4914
D156K-V61D	A/ A-B β-ribbon	3.05E + 08	56	148
G172R	B	8.75E + 05	1891	1103
G172R-D4N	B	2.0E + 07	902	611

Infectious titers for stocks of SINV chimera D72K-K159N and G172R-D4N mutants on BHK-21 cells were ~30 and 20-fold higher, respectively, than parental D72K and G172R mutants, and similar to the WT. The D156K-V61D mutant had ~ 500-fold higher infectious titer than D156K alone and ~10-fold higher than WT (Table 3). Furthermore, the additional mutations yielded a decrease in GE:PFU ratios *versus* their parental mutations and to values below the WT GE:PFU ratio suggesting higher infectivity (Table 3).

**Fig 7:**
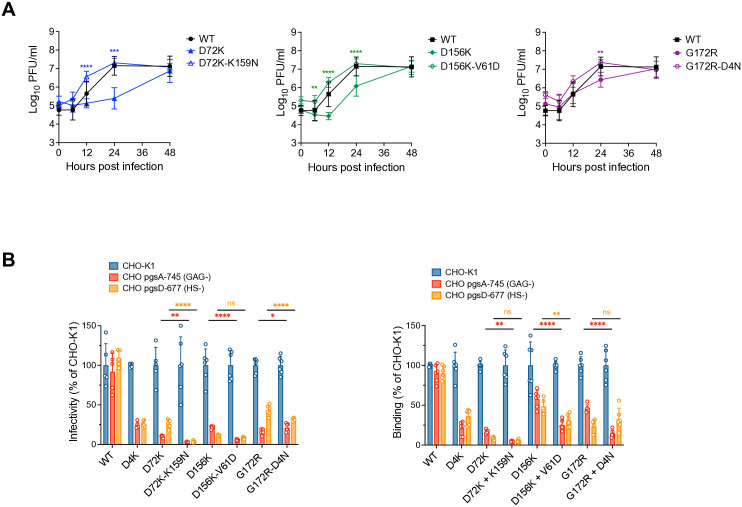
*In vitro* passage of downselected E2 mutants selected second mutations that increased the yield of virions while retaining HS binding phenotypes. SINV-WEEV E2 selected double mutants replication *in vitro* in Vero cells **(A)**. Vero cells were infected with equal genomes of the indicated LAV candidates corresponding to a multiplicity of infection equal to 1 PFU per cell for WT WEEV. Media were collected at the indicated times post infection, and infectious titers were determined by plaque assay on BHK-21 cells. **(B)** Relative infectivity and binding of the SINV-WEEV McM E2 selected double mutants compared to parental downselected mutants on CHO-K1, GAG-deficient pgsA-745 and HS-deficient pgsD-677 cells. For the infectivity assay, viruses were diluted and assayed for plaque formation on the indicated cell monolayers. GFP expressing foci were enumerated with a fluorescence microscope. For binding assays, diluted viruses at MOI of 5 were added to indicated cells and incubated for 45 min at 4 °C. Unbound virions were removed by washes with cold PBS, and ddPCR was used to assess the amount of bound viral particles. Percent infectivity and binding were normalized to CHO-K1, which was set to 100%. Data are combined from (A) two experiments performed in triplicate and (B) three experiments performed in duplicate. Error bars show SD; significance was estimated by (A) two-way ANOVA with Tukey’s post-hoc tests on log-transformed data, (B) one-way ANOVA with Bonferroni’s post-test.: * P < 0.05; ** P < 0.01, *** P < 0.001, **** P < 0.0001.

Growth curves were performed on Vero cells to evaluate the replication efficiency of the SINV chimera second site mutants in comparison with the original downselected mutations and WT SINV-WEEV. The double mutants replicated similarly to the WT and significantly more efficiently than the parental single mutants at 12 (P < 0.0001) and 24 hpi (P < 0.001) for D72K-K159N and D156K-V61D, at 6 (P < 0.01), 12 and 24 hpi (P < 0.0001) for D72K-K159N, and 24 hpi (P < 0.01) for G172R-D4N (Fig 7A). These data indicate a significant improvement in stability and/or infectivity of selected double mutants.

HS dependence of the infectivity and binding of the SINV chimera double mutants was evaluated similarly to the single mutants, using CHO-K1 pgsA-745 and pgsD-677 cells. Addition of the K159N mutation to D72K and V61D to D156K increased the dependence of infectivity (D72K-K159N: P < 0.01 in pgsA-745 and P < 0.0001 in pgsD-677; V61D-D156K: P < 0.0001 in pgsA-745, but non-significant in pgsD-677) and binding upon GAGs/HS (D72K-K159N: P < 0.01 in pgsA-745 and nonsignificant in pgsD-677; V61D-D156K: P < 0.0001 in pgsA-745 and P < 0.01 in pgsD-677) compared to single mutant viruses (Fig 7B). The D4N mutation, selected with E2 G172R, while decreasing the GE:PFU ratio, significantly increased the dependence of infectivity in pgsD-677 (P < 0.0001), but not in pgsA-745 and binding upon GAGs/HS (P < 0.0001 in pgsA-745) (Table 3 and Fig 7B), suggesting an impact on another aspect of the replication process.

### LAV candidate E2 mutations interfere with WEEV protein receptor interactions

**Fig 8:**
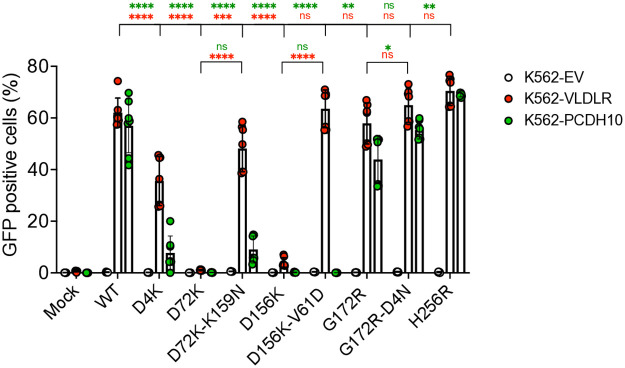
LAV candidates E2 protein mutations interfere with WEEV-protein receptor interactions. K562 cells stably expressing empty vector (EV), VLDLR or PCDH10 were inoculated with the indicated chimeric eGFP reporter wild-type or mutant SINV-WEEV at an MOI of 2. Infection levels were quantified by flow cytometry and expressed as percent infectivity. Data are combined from two experiments; one performed in triplicate and the other in quadruplicate. Error bars show SD; significance was estimated by two-way ANOVA with Tukey’s post-test: * P < 0.05; ** P < 0.01, *** P < 0.001, **** P < 0.0001.

During the performance of these studies, VLDLR, LDLR, and PCDH10 proteins were identified as mammalian entry receptors for WEEV [20,21,43,44], and the North American WEEV group A McM strain can use VLDLR or PCDH10 for cell entry [20,44]. Therefore, we tested the original downselected mutations and the second site mutations and WT WEEV McM for effects on enhancement of infectivity on K562 cells expressing VLDLR or PCDH10 receptors (Fig 8). All the originally downselected mutants showed reduced enhancement of infection over the empty vector cells compared with the WT virus. The D4K mutation resulted in partial reduction in infection enhancement for cell lines expressing either receptor, with a smaller effect observed for VLDLR *versus* PCDH10.

Mutants D72K and D156K exhibited a dramatic decrease (P < 0.0001) in infectivity enhancement for both receptors compared to the WT virus and little to no enhancement *versus* empty vector cells, suggesting significant disruption of the virus-receptor interaction concomitant with acquisition of HS utilization. However, second site mutations combined in the double mutants, D72K-K159N and D156K-V61D, largely restored the enhancement to WT levels on VLDLR expressing K562 cells, but not PCDH10 expressing cells. This suggests that improvement of protein receptor utilization was one phenotype selected with these second site mutations. The single mutant G172R showed minimal decrease in infectivity enhancement on both receptor expressing cell lines compared to the WT virus, and the second site D4N mutation increased the infectivity of the double mutant to the WT virus level for both receptors. We also tested the infectivity enhancement of the H256R mutant, originally selected through WT virus passage, which exhibited little HS infectivity dependence over the WT virus. This mutant showed significant enhancement of infectivity on both cell lines over the control cells and to slightly higher levels compared to the WT virus, significant on PCDH10 (P < 0.05), but not VLDLR expressing K562 cells.

### Candidate E2 mutant LAVs function as attenuated and immunogenic vaccine vectors

**Fig 9:**
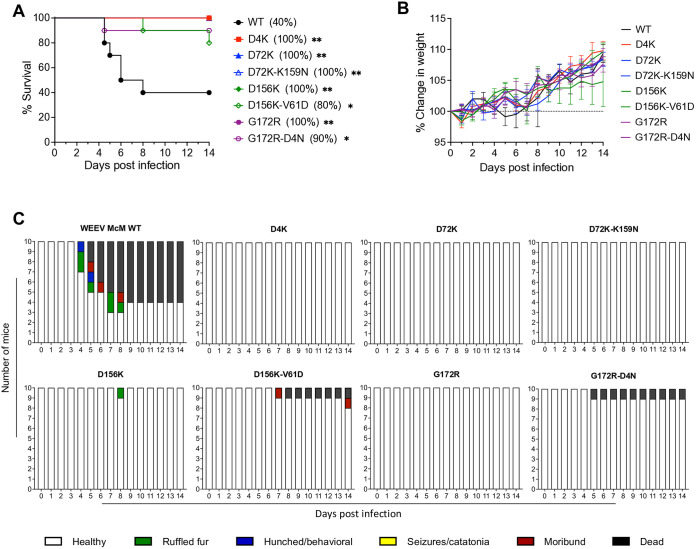
Mouse survival after subcutaneous candidate E2 mutant LAV infection. 4–6-week-old CD-1 mice were infected subcutaneously in rear footpad with the same dose of 10^3^ PFU of WT WEEV and WEEV E2 mutant LAV candidates. Experiments included 5 animals per group and were performed twice. Mice were monitored for survival and weight change for 14 days. **(A)** Survival. **(B)** Weight change. **(C)** Clinical signs. Error bars are standard error of the mean. The significance between survivals of mice infected with WT WEEV and mutants was estimated using the Log-rank (Mantel-Cox) test; *P < 0.05, **P < 0.01.

Female outbred CD-1 mice (4–6 weeks old) were infected with 10^3^ PFU of each WEEV virus subcutaneously and monitored daily for morbidity and mortality. All WEEV E2 mutant LAV candidates were attenuated compared to WT WEEV (Fig 9A and 9B). Furthermore, all downselected single mutants and the D72K-K159N double mutant yielded 100% survival (P < 0.01) and no signs of disease (Fig 9C). Double mutants D156K-V61D and G172R-D4N had 80% and 90% survival, respectively (P < 0.05) suggesting that the virulence of these viruses was increased *versus* the parental mutations but still lower than WT WEEV, possibly reflecting the increase in cell infectivity and protein receptor utilization (reduction in GE:PFU) conferred by the second site mutations.

**Fig 10:**
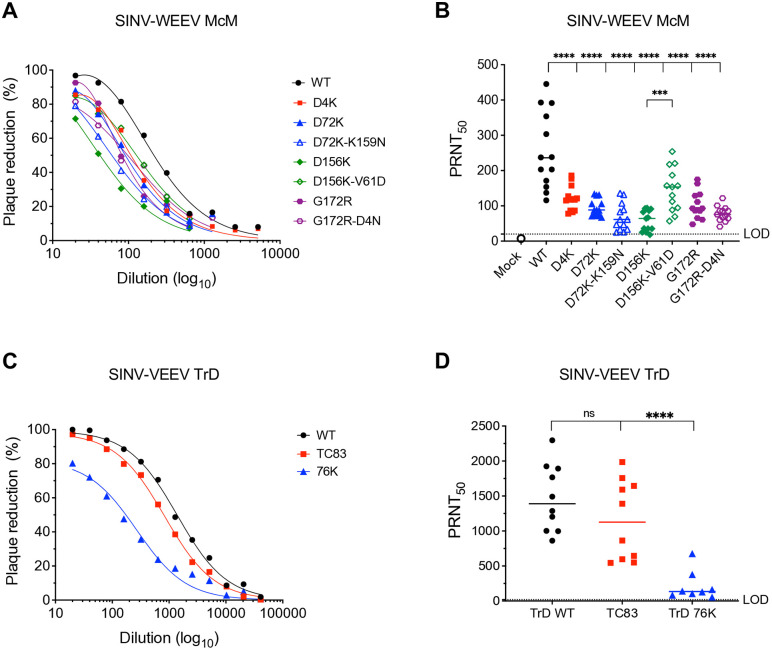
Downselected single and double E2 mutant LAV candidates induce neutralizing antibodies in mice. 4–week–old CD-1 mice were infected subcutaneously with equal genomes of indicated SINV-WEEV McM chimeric LAV candidates and control SINV-VEEV TrD viruses, equivalent to 5x10^3^ PFU (BHK-21) of WT SINV-WEEV McM and SINV-VEEV TrD viruses, respectively. Experiments included 3-5 animals per group and were performed twice for SINV-VEEV TrD controls and three times for SINV-WEEV McM LAVs. Serum samples were collected on day 21 post infection and screened for neutralization antibody levels by Vero cell plaque neutralization assay using a SINV-WEEV McM WT chimeric virus for LAV candidates and SINV-VEEV TrD WT – for controls. Sera were serially diluted 2-fold before reaction with the virus. Two independent dilutions of each sample were performed. **(A, C)** Nonlinear curve fits to dilution versus plaque reduction data. **(B, D)** Reciprocal PRNT_50_ values for each mouse were calculated as described in Materials and Methods. Significance of differences was determined by two-way ANOVA with Tukey’s post-test: * P < 0.05; ** P < 0.01, *** P < 0.001, **** P < 0.0001.

We then tested the generation of neutralization titer in mice using SINV chimeric viruses expressing the structural proteins of each mutant to eliminate any mortality that might occur with parental WEEV. Mice were infected with equal genomes of each of the vaccine candidates equivalent to 5x10^3^ PFU (BHK-21) for WT SINV-WEEV. Sera collected from the infected mice were tested against a SINV-WEEV WT chimera for neutralizing activity in a standard plaque reduction neutralization titer (PRNT) assay on Vero cells. Serum from the WT SINV-WEEV infected mice and mice infected with most of the candidate LAVs showed over 80% neutralization at 1:20 dilution of serum with the highest neutralization elicited by the WT SINV-WEEV chimera (97% neutralization, Fig 10A). D72K-K159N and D156K were the lowest with 79% and 71.5% neutralization, respectively.

Serum from mice infected with D4K, D72K, D156K-V61D and G172R neutralized SINV-WEEV WT similarly, with PRNT_50_ values from 1:95–1:146, which were significantly reduced compared to WT (1:257, P < 0.0001) (Fig 10B). Vaccination with D156K, D72-K159N and G172R-D4N produced lower PRNT_50_ values of 1:58, 1:68 and 1:78, respectively, compared to WT (P < 0.0001). Average PRNT_50_ values for the double mutant viruses D72K-K159N and G172R-D4N were not significantly different from parental single mutants. However, the D156K-V61D double mutant virus showed a significant increase in PRNT_50_ compared to its D156K parental mutant virus (P < 0.001). Thus, all HS binding viruses elicited lower neutralization titers than the non-HS binding WT WEEV parent.

To determine the effect of HS binding on neutralization titers elicited by another alphavirus with paired HS binding mutants, we also compared chimeras generated from the minimally HS binding VEEV WT Trinidad Donkey (TrD) strain structural proteins to two HS binding mutants of TrD. The TC83 vaccine strain, which has several charge-altering mutations in the E2 protein including: K7N, H85Y, T120R, and V192D, as well as a mutation L161I in the E1 protein [45], with the T120R primarily contributing to the HS binding phenotype [18]. Furthermore we evaluated an E2 E76K single-site mutant derived from *in vitro* passage of TrD [18]. These viruses exhibited 100%, 99% and 84% neutralization at 1:20 dilution of serum, respectively (Fig 10C). TrD WT, TC83 and E76K neutralized SINV-TrD WT with PRNT_50_ values of 1:1473, 1:1157 and 1:217 (P < 0.0001), respectively, indicating neutralization capacity is reduced with HS binding mutants of VEEV as well (Fig 10D).

**Fig 11:**
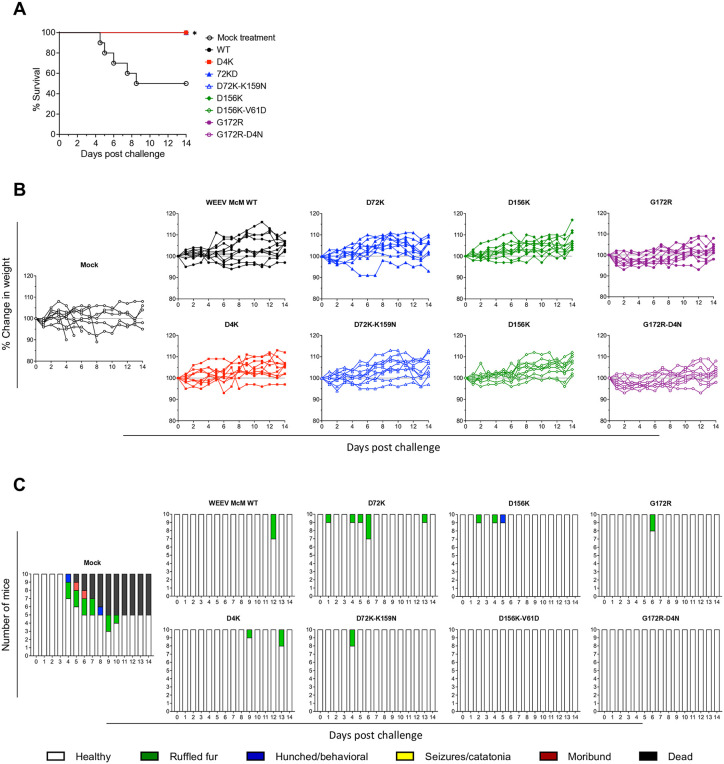
E2 mutant LAV candidates protect mice from parental WT WEEV McM s.c. challenge. CD-1 mice were challenged subcutaneously with 1x10^3^ PFU of parental WT WEEV McM virus on day 22 post primary SINV-WEEV-eGFP LAV candidates infection. Experiments included 5 animals per group and were performed twice. Mice were monitored for survival and weight change for 14 days. **(A)** Survival, **(B)** weight change, **(C)** clinical signs. The significance between survivals of WT WEEV McM challenged mice infected with WT WEEV and mutants was estimated using the Log-rank (Mantel-Cox) test; * P < 0.05.

Finally, we challenged the SINV-WEEV chimera and control (PBS) vaccinated mice 22 days after primary vaccination, s.c. with 1x10^3^ PFU of parental WT WEEV McM and monitored morbidity and mortality for 14 days (Fig 11A). In the control mock-vaccinated group 3 out of 5 mice died by day 8. The WT chimera and all vaccine candidate chimeras provided 100% protection from mortality with no significant weight loss after WEEV challenge (Fig 11B). Some mice vaccinated with the WT, single mutant vaccine candidates, and D72K-K159N, showed signs of disease but recovered by day 14 (Fig 11C). Mice vaccinated with double mutants D156K-V61D and G172R-D4N showed no signs of disease after WEEV challenge.

### Candidate E2 mutant LAVs replication in mice PLNs and brains

**Fig 12:**
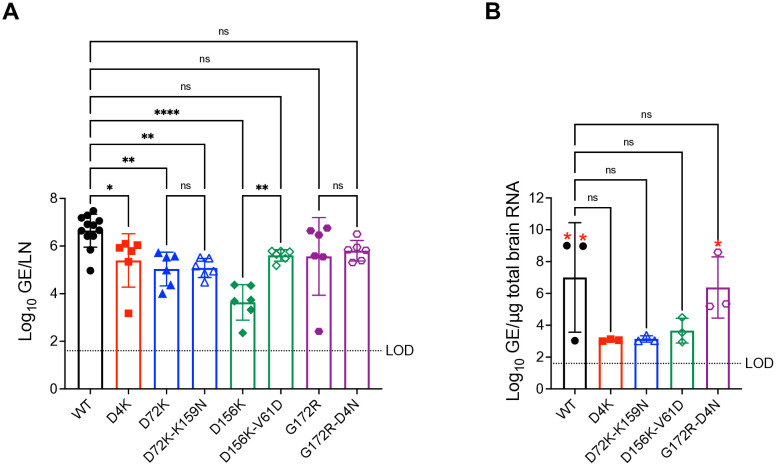
Replication of WEEV McM LAV candidates in popliteal lymph nodes and brains. CD-1 mice were infected with equal genome copies of the WEEV McM LAV candidates corresponding to 10^3^ PFU of WT WEEV. **(A)** Popliteal lymph nodes were collected at 8 hpi, and viral RNA levels were quantified by qRT-PCR using primers mapping to the WEEV nsP2 region. Data are presented as log_10_ genome equivalents per LN. **(B)** Brains were collected at 4 and 5 dpi, and viral RNA levels were quantified as in panel A. Each point represents an individual mouse (PLNs: n = 12 for WT and n = 6 for each mutant; brains: n = 3 per group). Each red asterisk indicates a mouse that died on or required euthanasia due to infection on day 4 (WT) and day 5 (G172R-D4N) after infection. Dotted line indicates the limit of detection (LOD) of the assay. Statistical comparisons to WT were performed on log_10_ transformed values using one-way ANOVA with Bonferroni’s post-test. *P < 0.05, **P < 0.01, ***P < 0.001, ****P < 0.0001.

To determine whether WEEV LAVs HS-dependent E2 protein mutations affects early *in vivo* dissemination, we quantified viral RNA levels in popliteal lymph nodes (PLNs) at 8 hours post-infection following footpad inoculation (Fig 12A). WT WEEV replicated efficiently at this early timepoint, with RNA loads ranging from 9.27 x 10^4^ to 3.01 x 10^7^ GE/LN (median 6.1 x 10^6^).

Several single mutants showed significantly reduced replication compared with WT. The single mutants D4K (*P < 0.05) and D72K (**P < 0.01) replicated to intermediate levels (1.48 x 10^3^ – 1.25 x 10^6^ and 1.03 x 10^4^ – 5.32 x 10^5^ GE/LN, respectively). The D156K mutant exhibited the strongest effect, producing only 2.24 x 10^2^ – 2.32 x 10^4^ GE/LN, representing a 3-log reduction compared with WT (****P < 0.0001). Double mutant D72K-K159N replicated to 7.12 x 10^4^ –3.34 x 10^5^ GE/LN, which was significantly lower than WT (**P < 0.01) and comparable to its parental D72K mutant. The D156K-V61D double mutant (median 5.09 x 10^5^ GE/LN), as well as G172R (1.65 x 10^6^ GE/LN) and G172R-D4N (6.79 x 10^5^ GE/LN), exhibited partial rescue and did not differ significantly from WT.

To assess CNS dissemination, we measured viral loads in brain tissue at 5 dpi, with samples from animals that succumbed earlier collected at the time of death. Two WT infected mice died on day 4 and one G172R-D4N infected mouse died on day 5, and these brains contained very high viral loads (WT: 1.01 x 10^9^ and 9.36 x 10^8^ GE/µg RNA; G172R-D4N: 3.92 x 10^8^ GE/µg RNA). Two other G172R-D4N infected mice that survived had lower viral loads ~10^5^ GE/µg RNA. In contrast, all remaining HS-dependent mutants and the third WT brain collected at 5 dpi showed extremely low CNS viral RNA levels (8.89 x 10^2^ – 3.09 x 10^4^ GE/µg RNA), often near the assay’s lower detection limit. Sequencing of WT and G172R-D4N brain isolates confirmed retention of engineered E2 mutations without additional or reverting mutations. These findings further support that the observed differences in attenuation arise from altered HS utilization rather than mutation instability.

Together, these results indicate that strongly HS-dependent LAV candidates exhibit restricted lymphoid replication and impaired CNS invasion, whereas the partially virulent G172R-D4N mutant retains WT-like dissemination.

## Discussion

WEEV has recently emerged in South America causing fatal infections in both humans and equines and represents a threat for further emergence in the Americas. This increases the need for effective antiviral countermeasures. In the current studies, we initially observed that the WEEV McM minimally bound and/or derived infectivity through engagement of HS receptors. Unlike our experience with other alphaviruses, passage of WT WEEV in BHK cells did not lead to acquisition of mutations that conferred efficient HS binding. We therefore used WEEV as an HS-negative background to develop an informed method for the generation of HS-binding and attenuated alphavirus vaccine candidates by inserting positively charged amino acid substitutions at similar positions in the E2 glycoprotein to those selected by cell culture amplification of other alphavirus strains and mapping them into a new cryo-EM reconstruction of the WEEV McM strain particles. We included several random negatively-positively charged mutations in different E2 domains as controls for non-targeted increases in the positive charge of the E2 glycoprotein. Finally, we used cell culture passage of mutant strains as a method for increasing the yield of particles in candidate vaccines to address scale-up issues in LAV production.

As part of these studies, a new Cryo-EM reconstruction of the WEEV McM strain was completed. The localized reconstruction of an asymmetric unit resolved the structure to a resolution of 3.2 Å, thus facilitating atomic model building. All four envelope protein heterodimers within the asymmetric unit could be fitted against the electron density map with confidence in COOT, except for the N-terminus serine residues and their side chains within the q3 trimeric spike and serine and isoleucine in the i3 spike. As the densities for the N-terminal residues were not well resolved, c-alpha and side chain fitting of these residues were conducted at a high contour level to generate one of the q3 spikes, which was later used for mapping the mutations. The N-terminal residues of the protein tend to be flexible/disordered, which could have possibly resulted in those densities being unresolved [46]. Additionally, the heterodimer occupies the quasi-equivalent environment, resulting in higher flexibility than in the strict i3 environment [47]. We have placed mutations created in the WEEV McM E2 glycoprotein into this new high-resolution structure as a guide for understanding the relationship of WEEV domain structure to host cell interaction.

As expected, passage of SINV-WEEV McM on BHK-21 fibroblasts selected for a positively charged amino acid substitution mutation, as has been observed with other alphaviruses [12]. Surprisingly, this mutation, while increasing binding and infectivity for BHK cells, did not appear to increase the HS dependence of WEEV infection and, therefore, may involve engagement of other types of virus receptors. However, we did not observe significant increase in infectivity enhancement on K562 cells overexpressing VLDLR, compared to the WT virus, but there was a small but significant increase (1.2-fold P < 0.05) in PCDH10 protein receptor utilization with this mutant. This result suggests that variation in the mechanisms of adaptation of different alphaviruses to cultured cell passage exists and that positive charge mutations in alphavirus glycoproteins should be examined for their effects upon virus engagement with receptors other than HS. Interestingly, this mutant was modestly attenuated *in vivo versus* WT WEEV suggesting that altering the efficiency of receptor engagement may be a generally attenuating factor *in vivo* regardless of the mechanism.

Placement of previously identified positively charged amino acid substitution mutations into the WEEV E2 glycoprotein resulted in an increase in either HS dependent infectivity, binding or both phenotypes in all cases except for the K254R mutation, including randomly selected positions in domain A, the A-B connector and in domain B. Yet most of the substitutions in sites previously found with other viruses exhibited an increased HS dependence to a greater degree than random controls suggesting that domain similarity between the viruses was responsible for the positioning of the positive charges in a more appropriate position for HS interaction. Since these all involved a negative to positive charge substitution, it can be concluded that changing the charge balance in the E2 glycoprotein, even at single randomly selected sites, can change the surface properties of the virion and can alter virus-cell interactions.

Based on the degree of HS binding and infectivity increase and level of attenuation in mice *versus* the WT WEEV, we downselected four mutants for further investigation. In addition to HS interactions, each of these mutations also affected VLDLR- and PCDH10-dependent infectivity, reducing it to varying levels. The most pronounced effect was observed for D72K and D156K mutations, which showed little to no enhancement of infectivity on K562-VLDLR or K562-PCDH10 overexpressing cells, suggesting abrogation of the protein receptor interaction. Recent cryo-EM studies mapped the contact residues mediating WEEV-VLDLR and WEEV-PCDH10 binding [48,49]. Three of the identified WEEV E2 contact residues for PCDH10 were H71, D156 and H157. Notably, D72 was also identified as a VLDLR contact residue [49]. Consistent with this report, our data show that mutations introducing a negative-to-positive charge at position 72, which is adjacent to H71, and at position 156, abrogate PCDH10-dependent infectivity. These mutations also abrogated VLDLR infectivity enhancement in our experiments, suggesting that similar positions are important for engagement of that receptor as well. Furthermore, these observations strengthen the evidence we recently described [50] for a linkage between E2 glycoprotein domains involved in binding to HS and protein receptors. While the increases in HS binding in this case are associated with a decrease in protein receptor utilization, with EEEV, reduction in E2 positive charge was similarly associated.

Our attempt to improve virus yields by passage of the four downselected mutants on BHK cells was successful with three of the four, yielding second site mutations for the D72K-K159N, G156K-V61D and G172R-D4N and the combination of both mutations further reduced the GE:PFU ratios for each mutant indicating improvement of virus yield or infectivity *versus* the single mutations. Notably, the K159N mutation in the D72K-K159N double mutant lies within the E2 β-ribbon connector region, the D72K mutation alone abrogated VLDLR-dependent infectivity, but introduction of the K159N mutation partially restored this interaction, suggesting that K159N may realign the local electrostatic environment or glycoprotein conformation to favor receptor compatibility. This is consistent with structural data showing that the β-ribbon connector contributes to receptor-induced conformational changes during alphavirus entry [48,49].

Similarly, the D156K-V61D double mutant exhibited a significant recovery of VLDLR-dependent infectivity. The V61D mutation lies within domain A of the E2 protein and is spatially adjacent to D156 on the surface of the spike trimer. Both residues reside near the base of the β-ribbon and are likely positioned within the same receptor-accessible patch. Given that D156 directly contacts PCDH10 [46], and that D72 and D156 flank a common receptor-binding cleft, it is plausible that V61D indirectly stabilizes local folding or charge distribution to support VLDLR interaction that was disrupted by D156K. It is worth noting that alphavirus receptor usage is thought to depend not only on direct contact residues but also on broader conformational epitopes [45], and these findings support a model in which second-site mutations can rescue receptor binding by altering such surfaces.

In the case of the G172R-D4N double mutant, D4N is located at the N-terminus of domain A, a region less well-characterized structurally but positioned on the exterior of the spike. G172R resides in domain B, and while it showed only moderate disruption of receptor usage as a single mutant, addition of D4N increased PCDH10 and VLDLR infectivity enhancement to wild-type levels. While D4 has not been previously implicated in receptor contact, this suggests that domain A–B coordination may also play a role in maintaining receptor-binding geometry. Given that both PCDH10 and VLDLR bind across multiple E2 domains [45,46], even subtle repositioning of surface loops may significantly affect binding efficiency.

Two of the three second site mutations did slightly increase virulence in mice *versus* parental mutations. These increases may be associated with more efficient interaction with protein receptors as evidenced by the K562 overexpressing cells experiment, although, the D72K-D159N virus was still highly attenuated so this is not invariably the case. However, the enhanced virulence may exclude the G156K-V61D and G172R-D4N viruses from further consideration as vaccine candidates unless combined with other mutations in a vaccine vector. The D4K mutation, which did not yield a second site mutation after passage, exhibited the lowest GE:PFU ratio of all original downselected mutants on BHK-21 and CHO-K1 cells and exhibited replication characteristics similar to WT WEEV on Vero cells, which may imply lowered selection pressure for a mutation that increases replication efficiency in this context. This virus, along with the D72K-K159N double mutant, was highly attenuated in mice and completely protective from mortality and signs of disease after a single immunization with a SINV chimeric virus and represents candidate mutations for inclusion in a WEEV LAV.

Consistent with these receptor-usage phenotypes, *in vivo* replication studies showed that HS-dependent mutations restrict WEEV spread at a very early stage. All HS-binding mutants exhibited substantially reduced viral RNA levels in popliteal lymph nodes at 8 hpi compared with WT. The most pronounced defects were observed for D156K and the second-site mutant D72K-K159N, which showed the lowest PLN replication. G172R and double mutants G172R-D4N and D156K-V61D showed partial rescue and did not differ significantly from WT. This early impairment in lymph node replication correlated with low viral RNA levels in the brain at 5 dpi for all HS-dependent mutants, whereas high-level CNS replication was observed only in mice that succumbed to infection with WT or the partially virulent G172R-D4N. These data indicate that significant neuroinvasion occurred exclusively in lethal infections. Together, these findings indicate that enhanced HS dependence limits initial lymphoid replication and restrict neuroinvasion, providing a mechanistic explanation for the strong attenuation observed *in vivo* while still permitting sufficient antigenic exposure to drive protective immunity.

Interestingly, all viruses exhibiting a high dependence upon HS for infection elicited lower neutralization titers than parental viruses when mice were immunized with equal genomes of the SINV chimeras, both with WEEV and VEEV. This suggests that either the HS binding mutants do not transit to the most effective immune inductive sites *in vivo,* that the HS binding viruses do not infect cells as efficiently *in vivo* as *in vitro* where their infectivity is usually higher that parental viruses, or that polyclonal antibody responses elicited by these viruses are not as efficient as those elicited by the parental strains at neutralization of the parental strains. The first explanation is suggested by several lines of evidence including the fact that TC83, which has a limited increase in HS dependence versus TrD, elicited higher neutralization titer than the highly HS dependent E76K mutant [18], the fact that replication of HS binding viruses in lymph nodes draining the infection sites is typically lower than non-binding parentals ([50], reviewed in [12]), and the fact that HS binding viruses appear to be cleared from sera by phagocytic cells more rapidly than non-HS binding viruses [51]. Yet, efficient HS binding is a common attenuation mechanism among safe and effective LAVs derived by cell passage [12] and utilization of these mutations in LAVs, in particular when combined with other well characterized mutations (e.g., [52]) appears justified.

Ultimately, the informed placement of putative HS binding mutations combined with *in vitro* selection for particle yield increases has potential for improving scale up for vaccine production, which has not been considered previously with LAVs. It should also be noted that yields and purity of non-propagative alphavirus replicon-based vaccines utilizing HS binding variants have been increased by high ionic strength washes during harvesting of particles and purification using positively charged column media [53,54]. Therefore, use of HS binding mutations in vaccine candidates has utility in multiple aspects of LAV design and scale-up.

## Materials and methods

### Ethics statement

All mouse studies were conducted in accordance with the recommendations outlined in the Guide for the Care and Use of Laboratory Animals of the National Research Council. All procedures were approved by the Institutional Animal Care and Use Committee of the University of Pittsburgh under protocols 20047334 and 23043041. Mice were monitored daily, and euthanasia was performed according to approved criteria based on body weight loss and clinical signs of morbidity to minimize animal suffering.

### Cell lines

Baby hamster kidney (BHK-21) (ATCC CCL-10) cells were maintained in RPMI-1640 medium (Corning) supplemented with 10% (v/v) donor bovine serum, 10% (v/v) tryptose phosphate broth, 2 mM L-glutamine, 100 U/ml penicillin, and 0.1 mg/ml streptomycin. Chinese hamster ovary (CHO-K1) (ATCC CRL-61) and CHO mutant cells psgA-745 (ATCC CRL-2242) and pgsD-677 (ATCC CRL-2244) were maintained in Ham’s F-12 medium (Corning) with 10% (v/v) fetal bovine serum, 2 mM L-glutamine, 100 U/ml penicillin, and 0.1 mg/ml streptomycin. Vero (ATTC CCL-81) cells were maintained in DMEM medium (Corning) with 10% (v/v) fetal bovine serum, 2 mM L-glutamine, 100 U/ml penicillin, and 0.1 mg/ml streptomycin. *Aedes albopictus* (C6/36) cells were maintained in MEM alpha (Corning) containing 10% (v/v) fetal bovine serum and no antibiotics. K562 (ATCC CCL-243) human lymphoblast cells expressing human VLDLR, empty vector (EV) [19] or human PCDH10 [20] were maintained in RPMI-1640 with 10% (v/v) heat-inactivated FBS, 100 U/ml penicillin, 0.1 mg/ml streptomycin, 25 mM HEPES (Corning), and 2 µg/mL puromycin (Mirus).

### Viruses

All viruses used in this study were generated from cDNA clones. The SINV TR339 E2-D60R/V61T mutant was derived after ten serial passages of wild-type TR339 in C6/36 cells, and the mutations were identified by Sanger sequencing. The cDNA clone of Sindbis virus strain TR339 has been previously described [25]. WEEV McMillan cDNA, derived from human isolate, was a gift from Kenneth Olson, Colorado State University [26]. The entire virus sequence was transferred into the modified pBR322 vector and T7 transcriptional promoter, that increased yields of viral RNA during in vitro transcription reactions [55]. Replication-competent SINV-WEEV-eGFP chimera contains the backbone of SINV strain TR339 and the structural protein genes of WEEV McMillan under control of the SINV subgenomic promoter [27,56]. WEEV and SINV-WEEV-eGFP E2 mutants were generated by site-directed mutagenesis using appropriate overlapping primers and Quikchange II KL SDM kit (Agilent) according to manufacturer’s guidelines. Virus stocks were generated by T7 RNA polymerase for WEEV and SP6 RNA polymerase for SINV-WEEV-eGFP *in vitro* transcription (mMessage mMachine) of linearized plasmid DNA followed by electroporation into BHK-21 cells. Supernatants were harvested at 20-24h post-electroporation, clarified and stored as aliquots at -80°C. Virus titers were determined by plaque assay on BHK-21. Viral genomes were quantified by RT- qPCR of RNA extracted from virus stocks, as described below.

### Viral titers quantification by RT-qPCR

Viral RNAs were extracted from cell culture supernatants using TRIzol according to the manufacturer’s instructions. M-MLV Reverse Transcriptase was used to generate cDNA and viral genomes were quantified by qPCR using the following primers and probes: SINV nsP2 RT (5’-GCGTAATACGACTCACTATACTGTCTTCGACGTAATCTGCATAC-3’), SINV nsP2 qPCR forward (5’-GTTACCAGCGGAAAGAAAGA-3’), T7 promoter qPCR reverse (5’-GCGTAATACGACTCACTATA-3’), SINV nsP2 TaqMan probe (5’-6-FAM/ AATTGAGGCCGACGTGCTAAGACTG/BHQ_1–3’). To determine the absolute quantity of viral RNA in experimental samples, a standard curve was generated by 10-fold serial dilutions of in vitro synthesized RNA encoding the SINV-WEEV genomes from 1x10^8^ to 1x10^1^ copies.

For tissue samples, popliteal lymph nodes and brains were collected and homogenized in TRIzol. Total RNA was extracted according to the manufacturer’s instructions, and RNA concentrations were measured by NanoDrop. Viral RNA was quantified by qPCR using the Reliance One-Step Multiplex Supermix (Bio-Rad) with the WEEV McM nsP2 primers: forward (5’-CTATAAGACTGTAAAGACTCAGG-3’), reverse (5’-ACCAGATCACCAGTTAGG-3’) and TaqMan probe (5’-6-FAM/ CGCACGAAA/ZEN/GTGTGTTAAGCGAGAA/3IABkFQ-3’), following the manufacturer’s recommended cycling conditions. Equal amounts of total RNA from each sample were used. Absolute genome copy numbers were calculated using the standard curve generated from 10-fold serial dilutions of in vitro–synthesized WEEV McM RNA (4x10^9^ to 4x10¹ copies). Viral RNA levels are reported as genome equivalents per LN or per μg of total brain RNA.

### *In vitro* passage and *in vivo* genetic stability

BHK cell monolayers were infected with 10^3^ PFU of indicated SINV-WEEV-eGFP McM WT or E2 mutant viral stocks, followed by incubation at 37°C for 20–24 h. Supernatants were harvested, titered and used for infection of a new BHK monolayer with a 1 : 1000 dilution until ten passages had been achieved. Virus genome RNA was extracted from the BHK cell passage supernatants by TRIzol technique according to the manufacturer’s instructions. To sequence mutations in E2, cDNA was generated using SuperScript IV VILO (Invitrogen) and the entire E2 gene was PCR amplified using Deep Vent polymerase (NEB) and primers: forward – 5’-CTTTCGAATGTCACGTTCCCATGC-3’; reverse – 5’-TGAAGGAATGACTGTGTGGAATTTGC-3’. The PCR fragments were subjected for sequencing (GENEWIZ). RNA extracted from tissues of infected mice were sequenced by amplifying the E2 gene with the aforementioned primer pairs and the following pairs: forward – 5’-AGTTACAGCGCTGTGCGTGC and reverse – 5’-ACCTGCACGTTCGACCAACGC-3’.

### Infection experiments

CHO-K1 cells, glycosaminoglycan (GAG)-deficient pgsA-745 and heparan sulfate (HS)-deficient pgsD-677 CHO cells were infected with serially diluted in PBS-1% DBS eGFP-expressing double-promoter SINV-WEEV E2 mutant viruses. Overlay was applied 1 h after infection, and a standard plaque assay was performed. Infectious foci were then quantitated after 24 h by fluorescence microscopy using Olympus CKX41 inverted microscope and plaques were enumerated as PFU per milliliter. Data is shown as the percent infectivity compared to the CHO-K1 controls determined for each virus.

K562 cells expressing control EV, VLDLR or PCDH10 protein receptors were inoculated with equal genomes of each viral stock corresponding to an MOI of 2 of WT SINV-WEEV eGFP in RPMI-1640 media containing 10% FBS. Cells were harvested at 16 h postinfection, fixed in 4% PFA washed in FACS buffer and subjected to analysis using an BD Fortessa flow cytometer and FlowJo software. Data is shown as the percentage GFP expressing cells.

### Virus binding assay

Binding reactions were performed as described previously [27] with some modifications. Cells were dissociated from plates with enzyme-free cell dissociation buffer (Gibco) and seeded in 24 well plate, 12 h before the assay. Virus inoculum was applied at MOI of 5 based on a seeded cell density. Cells were incubated with virus at MOI of 5 on ice for 40 min with gentle agitation and then washed four times with 1 ml of PBS containing 1% FBS. Binding reactions were performed in duplicate, and all experiments were repeated twice. Cells were collected in 500 μl of TRIzol, total RNA was purified, and viral genomes per sample were quantified by digital droplet (dd) dPCR (Bio Rad QX200) using a ddPCR Supermix for probes (no dUTP). Mmadhc gene, that exhibited high expression stability across distinct CHO cell lines [57] was used as an internal control for normalization of qPCR data. Primers and probes used are as follows: SINV nsP2 RT/reverse (5’-CTGTCTTCGACGTAATCTGC-3’), forward (5’-GTTACCAGCGGAAAGAAAGA-3’), TaqMan probe (5’-6-FAM/AATTGAGGCCGACGTGCTAAGACTG/BHQ_1–3’); Mmadhc RT/reverse (5’-TGAAACTCGTTCACATACTGAGC-3’), forward (5’-TCACCTCAATGGGACTGC-3’), TaqMan probe (5’-5HEX/CTTTGCCTGATGTTCTAGCAGAGCC/BHQ_1–3’).

### Analysis of viral replication in vitro

Replication kinetics of chimera SINV-WEEV E2 mutant viruses were compared in Vero cells. Confluent monolayers grown in 24-well plates were infected in triplicate with equal genomes of each viral stock corresponding to a multiplicity of infection (MOI) of 1 PFU per cell of WT SINV-WEEV in phosphate-buffered saline (PBS) supplemented with 1% FBS. After 1 h of incubation, 1 ml of Vero complete growth media was added to each well. At 0, 6, 12 and 24h post infection, cell culture medium was collected and replaced with fresh medium. Virus titers were determined in the harvested samples by plaque assay on BHK-21 cells.

### Heparinase II sensitivity assay

Heparinase assay was performed as previously described [34]. BHK-21 cells were incubated with various concentrations of Heparinase II (New England BioLabs) for 1 h at 37°C. Cells were thoroughly washed with PBS-1% FBS, infected with indicated chimeric eGFP-expressing viruses in triplicate for 1 h at 37⁰C and overlayed with immunodiffusion-grade agarose. At 24 hpi, GFP-expressing plaques were visualized by fluorescence microscopy.

### Purification of SINV-WEEV McM viral particles

BHK-21 cells grown to 80–90% confluency were infected with SINV/WEEV McM viral particles at a multiplicity of infection (MOI) of 5. The infected cells were incubated for 16–20 hours at 37°C in the presence of 5% CO_2,_ while monitoring for cytopathic effect (CPE). The harvested supernatant was then centrifuged and further clarified using a 0.22µm filter. The viral particles were precipitated with 12% W/V polyethylene glycol (PEG)-8000 and 150 mM NaCl at 4°C overnight. This overnight precipitation was followed by centrifugation at 2500 x g for 30 minutes, after which the pellets were resuspended in 1X PBS (pH 7.4). Further centrifugation at 3000 × g for 5 minutes was conducted to remove excess PEG. The clarified and resuspended viral particles were then subjected to a continuous 10–90% Optiprep (Iodixanol) gradient ultracentrifugation for 1.5 hours at 247,000 × g using a SW-41 rotor. The purified particles were buffer-exchanged in 1X PBS (pH 7.4) to a final concentration of 1–2 mg/ml for further cryo-EM experiments.

### Cryo-EM sample preparation and data collection

The purified virus sample was vitrified on an ultrathin glow-discharged Lacey carbon grids (400 mesh; Ted Pella) by plunge-freezing in liquid ethane using the Cryo-Plunge-3 system (Gatan). The samples were visualized on a 300kV Titan Krios G1 (Thermo Fisher) equipped with a Gatan K3 detector, and micrographs were recorded using Leginon [58] software. A defocus range of -0.6 to -2.0 μm, pixel size of 0.666Å, and an electron dose of 20e^-^/pixel/sec were used for data collection at a magnification of 64,000X.

### Cryo-EM data processing and localized reconstruction

The beam-induced motion correction of the movies, the contrast transfer function (CTF) estimation, and particle picking were done utilizing CryoSPARC [39]. The particles thus picked were subjected to reference-free 2D-classification followed by *de novo* model building. Utilizing this as a reference, the 3D reconstruction of the viral particles was generated using homogeneous refinement, imposing icosahedral symmetry. The gold-standard Fourier Shell Correlation (FSC) coefficient of 0.143 was used for the determination of the resolution of the reconstructed particles.

For localized reconstruction, a focused mask around the asymmetric unit was generated, which was then used as a starting point for localized reconstruction using CryoSPARC v4.0. A final 3.2Å locally refined map, thus generated, was used for a rigid body fitting of an asymmetric unit (PDB ID: 8DEE) using University of California, San Fransico (UCSF) ChimeraX [59]. The map and the fitted PDB coordinates were imported into COOT [41] to rebuild the sequence. The resulting coordinates were iteratively processed in COOT and refined against the locally reconstructed map in Phenix [42] using the cryo-EM real space refine function.

### Virulence of viruses in mice

Four-week-old female CD-1 mice (Jackson Laboratories) were infected subcutaneously in left hind footpad with 1x10^3^ BHK PFU of each virus in 10 ul of Opti-MEM. Mouse experiments included groups of 4–5 mice. Subcutaneous infections were performed at least twice with similar results. Mice were observed for 14 days after infection at 24h intervals to determine mean survival times and percent of mortality. General clinical signs of infection were assessed by visual inspection, as well as daily weighing of the animals. The visual scoring system used was as follows: “0” for no disease signs; “1” for ruffled fur; “2” for mild behavioral abnormalities including altered mobility, lethargy; “3” for more severe behavioral disturbances including ataxia; “4” for paralysis or seizures. At designated times post-infection, mice were sacrificed via isoflurane overdose, perfused with PBS supplemented with 1% donor calf serum, followed by tissue harvesting directly into trizol. Tissues were homogenized and used for RNA extraction and subsequent qRT-PCR.

### Antibody neutralization assay

Serum samples from CD-1 mice immunized with the LAV candidates were collected on day 21 and heat inactivated at 56°C for 30min. The serum was serially diluted (2-fold dilutions) and incubated with ~100 PFU of WT SINV-WEEV-eGFP for 1h at 37˚C. Anti-WEEV ascites serum (ATCC) was used as a positive control. After incubation, Vero cells were infected in 6 well plates for 1h at 37°C in a plaque assay. After overlay with agarose immunodiffusion grade (MP Biomedicals), plates were incubated for 3 days followed by overlay with neutral red for at least 6h to count plaques. Percent neutralization was calculated based on the number of plaques in each serum dilution compared to the number of plaques in non-antibody treated control wells. A best fit non-linear curve was used to calculate the 50 percent reduction dilution (GraphPadPrism). Neutralization data was analyzed and PRNT50 calculated using Graphpad PRISM and the asymmetric sigmoidal 5PL standard curve fit (confidence limit 95%).

### Quantification and statistical analyses

Statistical significance was determined using Prism software (Version 10.1, GraphPad). Comparisons indicated in figure legends were determined by one-way analysis of variance with Turkey’s multiple-comparison test of log-transformed data or two-way analysis of variance with multiple comparisons using the Bonferroni method. I*n vivo* survival experiments were analyzed by Mantel-Cox log rank analysis. The statistical tests, number of independent experiments, and number of experimental replicates are indicated in the Figure legends.

## Supporting information

S1 Table E2 positive charge substitution mutations. Amino acid alignment of the indicated strains WEEV, EEEV, SINV, CHIKV, VEEV, GETV wild type and mutant E2 glycoprotein. Substitution mutations to positively charged residues are shown in bold red, corresponding wild type residues – bold black. Related to Table 1.
(PDF)

S1 Fig Cell culture passage of SINV-WEEV downselected E2 mutants. SINV-WEEV-eGFP E2 downselected mutants virus stocks were serially passaged 10 times on BHK-21 fibroblasts. 24 h after each passage, the quantity of infectious virions was titered on BHK-21 fibroblasts by standard plaque assay.
(TIF)

S2 Fig HS staining of CHO cell lines. Immunofluorescence staining of CHO-K1 and two mutant CHO cell lines: GAG-deficient pgsA-745 and HS-deficient pgsD-677 using anti-HS (AMSBIO F58-10E4) or isotype control (BD Biosciences G155-228). Cell staining was analyzed by flow cytometry using a BD LSRFortessa and FlowJo software.
(TIF)

S1 Data Raw data for figures 1A, 1C, 4, 7A, 9B, 10A, 10C, 11B and S1. (XLSX)
