# Supplementary material for: Informed mutation of western equine encephalitis virus to heparan sulfate binding: Implications for rational design of alphavirus live attenuated vaccines
Source: PLoS Pathog. 2026 Feb 9;22(2):e1013941. doi: 10.1371/journal.ppat.1013941 (PMC12912690; doi:10.1371/journal.ppat.1013941)
Supplement: S1 Table — Amino acid alignment of the indicated strains WEEV, EEEV, SINV, CHIKV, VEEV, GETV wild type and mutant E2 glycoprotein. Substitution mutations to positively charged residues are shown in bold red, corresponding wild type residues – bold black. Related to Table 1. (PDF) [file ppat.1013941.s001.pdf]

Virus<br>E2 WT/Mutant	E2 Sequence	Accession #	Reference
WEEV McM [a]	1 **S**ITDDFTLTS 10	(GQ287640)	[21]
WEEV McM E2-S1R	1 **R**ITDDFTLTS 10		
SINV AR339 E2-S1R	1 **R**VTDDFTLTS 10		
SINV AR339	1 **S**VTDDFTLTS 10	(CS227854)	
WEEV McM	1 SIT**D**DFTLTS 10		[15]
WEEV McM E2-D4K	1 SIT**K**DFTLTS 10		
VEEV TrD E2-E4K	1 STE**K**LFKEYK 10		
VEEV TrD	1 STE**E**LFKEYK 10	(L01442)	
WEEV McM	56 AGTA**DV**TKFR 65		(This manuscript)
WEEV McM E2-D60R/V61T	56 AGTA**RT**TKFR 65		
SINV TR339 E2-D60R/V61T	56 SGAA**RT**NKYR 65		
SINV TR339	56 SGAA**DV**NKYR 65	(OR882082)	
WEEV McM	65 RYMSY**D**HDHD 74		[21]
WEEV McM E2-D70K	65 RYMSY**K**HDHD 74		
SINV TR339 E2-E70K	65 RYMSL**K**QDHT 74		
SINV TR339	65 RYMSL**E**QDHT 74	(OR882082)	
WEEV McM	68 SYDH**D**HDIKE 77		[31]
WEEV McM E2-D72K	68 SYDH**K**HDIKE 77		
EEEV TX95 E2-T72K	68 MNGK**K**QKSIK 77		
EEEV TX95	68 MNGK**T**QKSIK 77	(AF159555)	
WEEV McM E2	70 DHDH**D**IKEDS 79		[30]
WEEV McM E2-D74K	70 DHDH**K**IKEDS 79		
EEEV FL93-939	70 GKTQ**K**SIKID 79	(EF151502)	
WEEV McM	73 HDIK**E**DSMEK 82		
WEEV McM E2-E77K	73 HDIK**K**DSMEK 82		
EEEV FL 93-939	73 QKSI**K**IDNLH 82	(EF151502)	[30]
VEEV TrD E2-E76K	73 TIK**K**IPLHQV 82		[15]
VEEV TrD	73 TIK**E**IPLHQV 82	(L01442)	
WEEV McM	74 DIKE**D**SMEKI 83		[12]
WEEV McM E2-D78K	74 DIKE**K**SMEKI 83		
CHIKV-LR E2-E79K	74 MPADA**K**RAGL 83		
CHIKV-LR	74 MPADA**E**RAGL 83	(DQ443544)	
WEEV McM	77 EDSM**E**KIAIS 86		[12]
WEEV McM E2-E81R	77 EDSM**R**KIAIS 86		
CHIKV-LR E2-G82R	77 DAERA**R**LFVR 86		
CHIKV-LR	77 DAERA**G**LFVR 86	(DQ443544)	

WEEV McM<br>WEEV McM E2-S114R<br>SINV AR339 E2-S114R<br>SINV AR339	110 SVTV**S**ITSGA 119<br>110 SVTV**R**ITSGA 119<br>110 SVTV**R**IVSSN 119<br>110 SVTV**S**IVSSN 119	(CS227854)	[21]
WEEV McM<br>WEEV McM E2-D156K<br>EEEV FL93-939	156 CHVY**D**RLKET 165<br>156 CHVY**K**RLKET 165<br>152 RYTH**K**RADQG 161	(EF151502)	[32]
WEEV McM<br>WEEV McM E2-E160K<br>CHIKV-LR E2-S159R<br>CHIKV-LR	156 DRLK**E**TSAGY 165<br>156 DRLK**K**TSAGY 165<br>156 YVQ**R**TAATTE 165<br>156 YVQ**S**TAATTE 165	(DQ443544)	[12]
WEEV McM<br>WEEV McM E2-G172R<br>SINV AR339 E2-G172R<br>SINV AR339	168 MHRP**G**PHAYK 177<br>168 MHRP**R**PHAYK 177<br>168 MHRP**R**PHAYT 177<br>168 MHRP**G**PHAYT 177	(CS227854)	[33]
WEEV McM<br>WEEV McM E2-E182K<br>WEEV CBA/87 E2-E182K<br>WEEV CBA/87	178 SYLK**E**ASGEV 187<br>178 SYLK**K**ASGEV 187<br>178 SYLE**K**ASGEV 187<br>178 SYLE**E**ASGEV 187	(DQ432026)	[34]
WEEV McM<br>WEEV McM E2-G209R<br>SINV AR339 E2-G209R<br>SINV AR339<br>VEEV TrD E2-E209K<br>VEEV TrD	205 DYST**G**IVSTQ 214<br>205 DYST**R**IVSTQ 214<br>205 DYKT**R**TVSTR 214<br>205 DYKT**G**TVSTR 214<br>205 TKIS**K**TINKT 214<br>205 TKIS**E**TINKT 214	(CS227854)<br><br>(L01442)	[33]<br><br>[15]
WEEV McM<br>WEEV McM E2-K254R<br>GETV E2-K253R<br>GETV	249 HSVQG**K**LHIP 258<br>249 HSVQG**R**LHIP 258<br>248 LSRKG**R**VHVP 257<br>248 LSRKG**K**VHVP 257	(MZ736801)	[29]
WEEV McM<br>WEEV McM E2-D109K	105 CPPG**D**SVTVS 114<br>105 CPPG**K**SVTVS 114		Control
WEEV McM<br>WEEV McM E2-E138K	134 FVGR**E**EYLFP 143<br>134 FVGR**K**EYLFP 143		Control
WEEV McM<br>WEEV McM E2-D205K	201 CKCG**D**YSTGI 210<br>201 CKCG**K**YSTGI 210		Control

[a] WEEV McM (GenBank accession number GQ287640, shown once).
