# Supplementary figures and images for: Informed mutation of western equine encephalitis virus to heparan sulfate binding: Implications for rational design of alphavirus live attenuated vaccines
Source: PLoS Pathog. 2026 Feb 9;22(2):e1013941. doi: 10.1371/journal.ppat.1013941 (PMC12912690; doi:10.1371/journal.ppat.1013941)

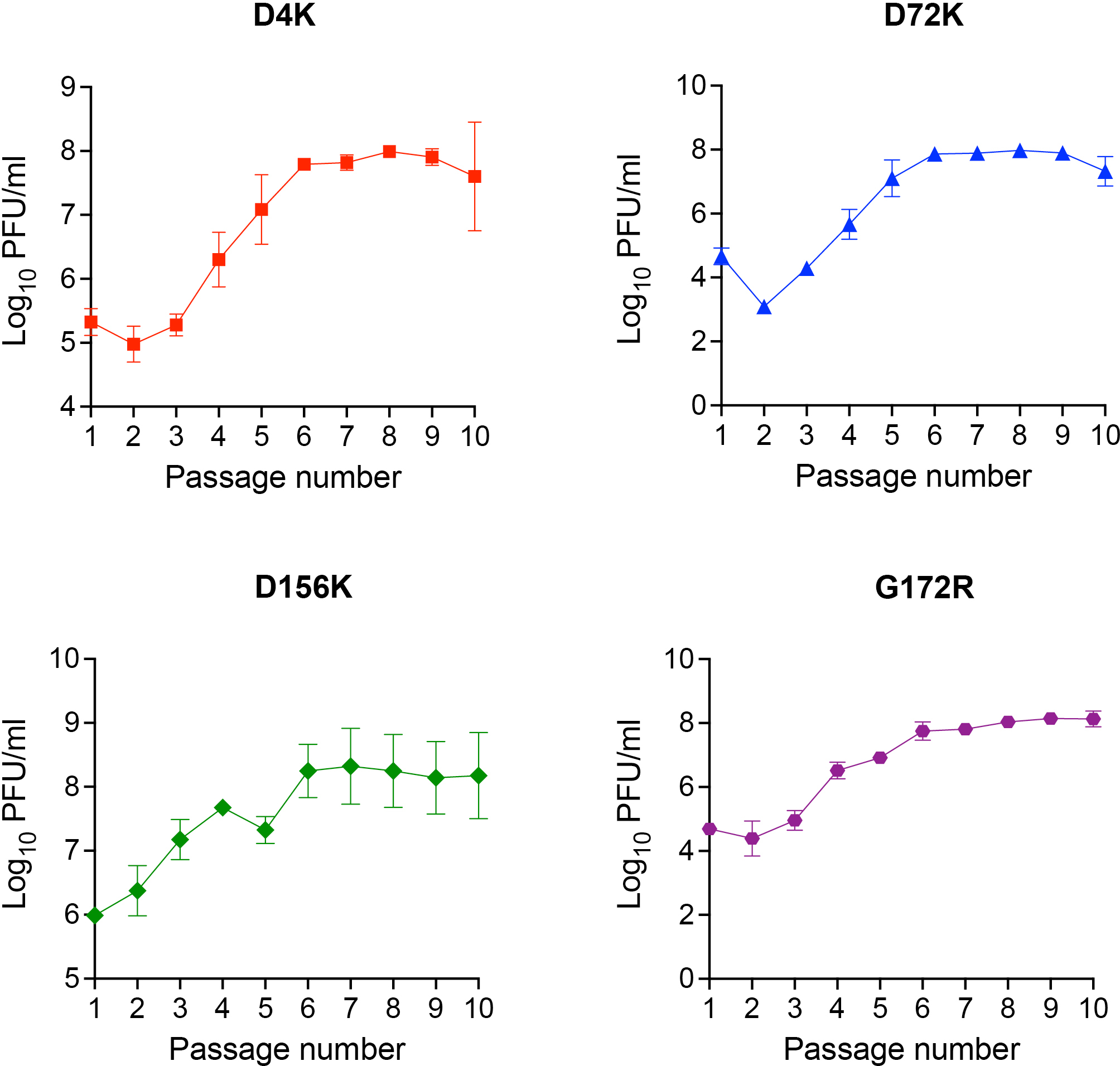

Supplement: S1 Fig — SINV-WEEV-eGFP E2 downselected mutants virus stocks were serially passaged 10 times on BHK-21 fibroblasts. 24 h after each passage, the quantity of infectious virions was titered on BHK-21 fibroblasts by standard plaque assay. (TIF) [file ppat.1013941.s002.tif]

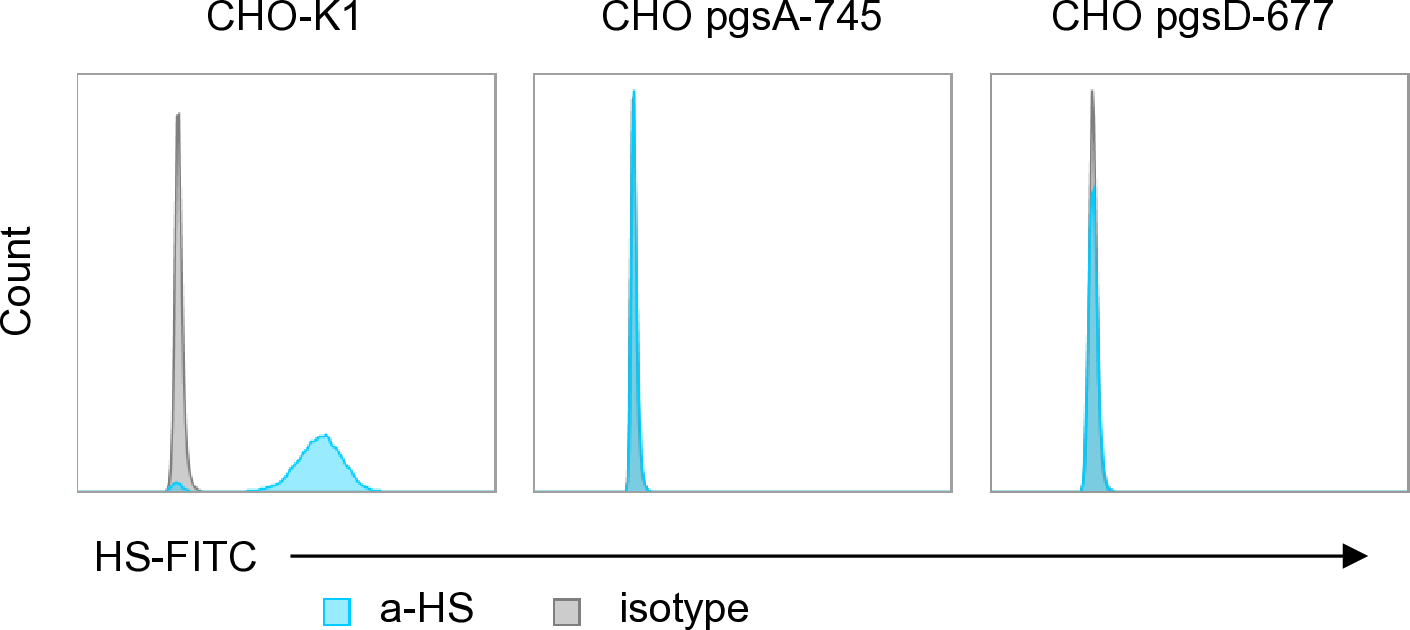

Supplement: S2 Fig — Immunofluorescence staining of CHO-K1 and two mutant CHO cell lines: GAG-deficient pgsA-745 and HS-deficient pgsD-677 using anti-HS (AMSBIO F58-10E4) or isotype control (BD Biosciences G155-228). Cell staining was analyzed by flow cytometry using a BD LSRFortessa and FlowJo software. (TIF) [file ppat.1013941.s003.tif]
